# Succession and determinants of the early life nasopharyngeal microbiota in a South African birth cohort

**DOI:** 10.1186/s40168-023-01563-5

**Published:** 2023-06-05

**Authors:** Shantelle Claassen-Weitz, Sugnet Gardner-Lubbe, Yao Xia, Kilaza S. Mwaikono, Stephanie Harris Mounaud, William C. Nierman, Lesley Workman, Heather J. Zar, Mark P. Nicol

**Affiliations:** 1 Division of Medical Microbiology, Department of Pathology, Faculty of Health Sciences, University of Cape Town, Cape Town, South Africa; 2 Department of Statistics and Actuarial Science, Faculty of Economic and Management Sciences, Stellenbosch University, Stellenbosch, South Africa; 3 Marshall Centre, Division of Infection and Immunity, School of Biomedical Sciences, University of Western Australia, Perth, Australia; 4 Center for Artificial Intelligence and Machine Learning, School of Science, Edith Cowan University, Joondalup, Australia; 5 Computational Biology Group and H3ABioNet, Department of Integrative Biomedical Sciences, University of Cape Town, Cape Town, South Africa; 6 Department of Science and Laboratory Technology, Dar Es Salaam Institute of Technology, Dar Es Salaam, Tanzania; 7 J. Craig Venter Institute, Rockville, MD USA; 8 Department of Paediatrics and Child Health, Red Cross War Memorial Children’s Hospital, Cape Town, South Africa; 9 SAMRC Unit on Child & Adolescent Health, University of Cape Town, Cape Town, South Africa; 10 Institute of Infectious Disease and Molecular Medicine, Faculty of Health Sciences, University of Cape Town, Cape Town, South Africa

**Keywords:** 16S rRNA gene, Birth cohort, Microbiome, High-throughput sequencing, Infant, Longitudinal, Low- and middle-income country, Microbiota, Nasopharyngeal, Upper respiratory tract

## Abstract

**Background:**

Bacteria colonizing the nasopharynx play a key role as gatekeepers of respiratory health. Yet, dynamics of early life nasopharyngeal (NP) bacterial profiles remain understudied in low- and middle-income countries (LMICs), where children have a high prevalence of risk factors for lower respiratory tract infection. We investigated longitudinal changes in NP bacterial profiles, and associated exposures, among healthy infants from low-income households in South Africa.

**Methods:**

We used short fragment (V4 region) 16S rRNA gene amplicon sequencing to characterize NP bacterial profiles from 103 infants in a South African birth cohort, at monthly intervals from birth through the first 12 months of life and six monthly thereafter until 30 months.

**Results:**

*Corynebacterium* and *Staphylococcus* were dominant colonizers at 1 month of life; however, these were rapidly replaced by *Moraxella-* or *Haemophilus*-dominated profiles by 4 months. This succession was almost universal and largely independent of a broad range of exposures. Warm weather (summer), lower gestational age, maternal smoking, no day-care attendance, antibiotic exposure, or low height-for-age *z* score at 12 months were associated with higher alpha and beta diversity. Summer was also associated with higher relative abundances of *Staphylococcus*, *Streptococcus*, *Neisseria*, or anaerobic gram-negative bacteria, whilst spring and winter were associated with higher relative abundances of *Haemophilus* or *Corynebacterium*, respectively. Maternal smoking was associated with higher relative abundances of *Porphyromonas*. Antibiotic therapy (or isoniazid prophylaxis for tuberculosis) was associated with higher relative abundance of anerobic taxa (*Porphyromonas*, *Fusobacterium*, and *Prevotella*) and with lower relative abundances of health associated-taxa *Corynebacterium* and *Dolosigranulum*. HIV-exposure was associated with higher relative abundances of *Klebsiella* or *Veillonella* and lower relative abundances of an unclassified genus within the family Lachnospiraceae.

**Conclusions:**

In this intensively sampled cohort, there was rapid and predictable replacement of early profiles dominated by health-associated *Corynebacterium* and *Dolosigranulum* with those dominated by *Moraxella* and *Haemophilus*, independent of exposures. Season and antibiotic exposure were key determinants of NP bacterial profiles. Understudied but highly prevalent exposures prevalent in LMICs, including maternal smoking and HIV-exposure, were associated with NP bacterial profiles.

Video Abstract

**Supplementary Information:**

The online version contains supplementary material available at 10.1186/s40168-023-01563-5.

## Background

A growing body of evidence describes the importance of the microbiota of the respiratory tract as gatekeepers of respiratory health [1, 2]. Bacteria colonizing the nasopharynx play a key role in respiratory tract infection (RTI) in children, by protecting the airway lining against air-transmitted pathogenic infections [3]. Commensals such as *Corynebacterium* and *Dolosigranulum* may benefit the ecosystem balance and respiratory health by excluding pathogenic bacteria [4–7]. However, the nasopharynx is also the natural niche for several potentially pathogenic species (pathobionts), including *Streptococcus*, *Haemophilus*, and *Moraxella* species.

Data from high-income settings indicate that early life nasopharyngeal (NP) bacterial communities transition from profiles dominated by *Staphylococcus* early in life towards *Corynebacterium-* and *Dolosigranulum-*enriched profiles, followed by enrichment with *Moraxella* later in infancy [8]. Temporal dynamics of these developmental stages could be important for respiratory health. For example, early enrichment with *Moraxella* and oral species, such as *Prevotella*, has been associated with an increased susceptibility to RTI during the first year of life [7]. Similarly, early life bacterial profiles dominated by *Haemophilus* or *Streptococcus* have been associated with respiratory virus infection and increased risk of bronchiolitis during infancy [9–11].

Exposures such as mode of delivery, feeding practices, and antimicrobials shape NP bacterial communities and may impact long-term respiratory health [4, 7]. For example, when compared to infants born by vaginal delivery, cesarean-section delivery has been associated with delayed succession of NP bacterial profiles and reduced colonization with health-associated taxa such as *Corynebacterium* and *Dolosigranulum* [12]. The protective role of breastfeeding against lower respiratory tract infection (LRTI) may be related, in part, to the presence of *Corynebacterium* spp. in breast milk [13], which could enhance the growth of commensals such as *Dolosigranulum pigrum* and protect against pathobionts such as *Staphylococcus* [14, 15]. Reduced relative abundance of *Corynebacterium* and shifts in NP bacterial community profiles have been associated with antibiotic administration [7, 16]. Other factors modulating early life NP bacterial profiles, also associated with LRTI, include indoor air pollution [17], tobacco smoke exposure [17], and seasonal changes [18, 19].

Data on longitudinal changes in NP bacterial profiles early in life, and factors influencing these profiles remain scarce—particularly from low- and middle-income countries (LMICs) [4, 7, 8, 12, 14, 16, 18, 20, 21]. Children in LMICs have a high prevalence of risk factors for LRTI. For example, malnutrition and HIV exposure have been associated with more severe LRTI and poorer outcomes [22–24]. Other risk factors for LRTI, common in LMICs, include short duration of exclusive breastfeeding [25], tobacco smoke exposure [22], indoor air pollution [26], lack of immunization, and suboptimal clinical care [26, 27]. Nonetheless, the impact of these exposures on early life NP bacterial communities in infants from LMICs is not well studied.

We therefore characterized succession of NP bacterial communities from children without LRTI living in a low-income setting at monthly intervals from birth for 12 months and six monthly thereafter until 30 months of life. We investigated how host and environmental factors influenced bacterial diversity and community composition.

## Methods

### Study setting

This study was nested within the Drakenstein Child Health Study (DCHS) [28], a birth cohort in South Africa, which longitudinally followed mother–child dyads through childhood to investigate the impact of early life exposures on child health [28]. Enrolment of consenting pregnant women (> 18 years of age) took place during their second trimester at public sector primary health care clinics. All births and hospital care occurred at Paarl Hospital (60 km outside Cape Town, South Africa), while all children received primary health care at these clinics [28].

The local community of approximately 200,000 people is of low socioeconomic status, and most residents live in informal housing and crowded conditions [28]. Participants experience high levels of unemployment, food insecurity, and other poverty-related exposures including tobacco smoke exposure, and indoor air pollution, as described [29, 30]. More than 90% of the population access health care, including antenatal services and HIV treatment and prevention of mother-to-child transmission programs (PMTCT), in the public sector [28]. LRTI [31], tuberculosis [32], and HIV exposure (HIV-uninfected infants born to HIV-infected mothers) [29] are common among children enrolled in the DCHS.

Participants included in this substudy had NP specimens collected at two weekly intervals during the first year of life [28], with additional 6 monthly study visits from 12 to 30 months [28].

### Measures

Antenatal ultrasound from the second trimester was used to calculate gestational age at delivery. Preterm was defined as < 37 weeks of gestation. Birth weight, length, and head circumference were measured at the time of delivery. Weight-for-age *z* (WAZ) scores at birth and at 12 months were calculated using the revised Fenton preterm growth charts [33, 34]. Low- and high-WAZ scores were defined as scores less and greater than 1 standard deviation (SD) below the mean weight-for-age value, respectively. Using previously published classifications of South African seasonal patterns, “birth season” and “specimen collection season” were categorized into autumn (April to May), winter (June to August), spring (September to November), and summer (December to March) [35, 36]. Feeding practices were longitudinally reported through infancy.

Maternal smoking was self-reported antenatally and 10 weeks postnatally. Monthly household income and maternal educational attainment were self-reported.

Children who experienced a LRTI were excluded from this analysis. Tuberculin skin tests were done six monthly and isoniazid (INH) prophylaxis provided at primary health clinics to those testing positive.

Maternal HIV infection was confirmed in pregnancy; all HIV-infected mothers received antiretroviral therapy (ART) as per local guidelines [37]. HIV-exposed children were tested for HIV by polymerase chain reaction (PCR) at 6 weeks, enzyme-linked immunosorbent assay (ELISA) or rapid antibody testing at 9 months, and rapid antibody testing at 18 months, as per guidelines [37]. HIV-exposed children who tested negative for HIV were classified as HIV-exposed, uninfected.

### Specimen collection and selection for 16S rRNA gene amplicon sequencing

NP flocked swabs (FLOQSwab™, Copan Diagnostics, CA, USA) were collected and immediately suspended in PrimeStore® Molecular Transport medium (Longhorn Vaccines & Diagnostics, Bethesda, MD, USA), transported on ice and stored at −80 °C until further processing. We included NP specimens collected at monthly intervals (using a window of ± 15 days) during the first year of life and then at 18, 24, and 30 months of life (using a window of ± 60 days).

### 16S rRNA gene amplicon library preparation and sequencing

Each sequencing run consisted of four 96-well plates (384 reactions). A comprehensive set of sequencing controls was included alongside NP specimens on each 96-well plate [38] (Supplementary material, Section A, Fig. S1). Nucleic acid extraction steps have been described in detail elsewhere [38, 39]. Briefly, we transferred 400 µl of homogenized NP specimen to ZR BashingBead™ Lysis Tubes containing 0.5 mm bashing beads (catalogue no. ZR S6002-50, Zymo Research Corp., Irvine, CA, USA) for mechanical lysis at 50 Hz for 5 min using the TissueLyser LT™ (Qiagen, FRITSCH GmbH, Idar-Oberstein, Germany). We loaded 250 μl of the supernatant to the QIAsymphony® SP instrument (Qiagen, Hombrechtikon, Switzerland) for automated nucleic acid extraction using the DSP Virus/Pathogen Mini Kit® (catalogue no. 937036, Qiagen GmbH, Hilden, Germany) [38] with an elution volume of 60 µl. We quantified total 16S rRNA gene copy numbers from each nucleic acid extract using quantitative polymerase chain reaction (qPCR) [18]. Amplicon library preparation steps have been described in detail elsewhere [38, 39] (Supplementary material, Section A). We sequenced the libraries on the Illumina® MiSeq™ platform using the MiSeq Reagent Kit v3 (600-cycle) Reagent Cartridge (Illumina, San Diego, CA, USA).

A detailed description of the bioinformatics approach is provided in Supplementary material, Section A. In brief, we first assessed the quality of demultiplexed paired-end reads via FastQC [40] and MultiQC [41]. We then used the DADA2 pipeline [42] (wrapped in the Nextflow algorithm [43]) to filter and trim reads, infer amplicon sequence variants (ASVs), and assign taxonomy to ASVs. We assigned taxonomy to each of the ASVs using the RDP [44] classifier implementation for DADA2 [45] and SILVA version 138 [46] (Supplementary Tables 1 and 2). We removed ASVs classified as Eukaryota and ASVs with unassigned taxonomy at Kingdom level from the dataset.

### Participant selection for downstream analyses

We used a step-wise in silico quality control approach [38] to ensure the inclusion of high-quality gene amplicon data (Supplementary material, Section A). We subsequently excluded HIV-infected participants (*n* = 1) and participants with ≥ 4 missing NP specimens from the first year of life.

### Statistical analyses

Details of statistical methods are described in Supplementary material, Section A. We used R software version 3.6.3 and RStudio software version 1.3.1056 for data analysis and visualization [47, 48]. We applied a one-way analysis of variance (ANOVA) to compare Shannon diversity indices [49] between timepoints and implemented Tukey’s Honest Significant Difference test to compare each pair of timepoints simultaneously pairwise. We computed Aitchison distances [50, 51] between specimens at each of the timepoints and across all timepoints. We used principal coordinate analysis (PCoA) plots to visually represent between-specimen beta diversity with 90%-bags [52] enclosing the inner 90% of the observations from each of the timepoints under study.

We used the R function [gm] in the package *robCompositions* [53] to calculate compositional mean relative abundances [54, 55] and the R function [barplot] in base R to generate compositional mean relative abundance barplots. Bootstrap confidence intervals of compositional mean relative abundances were computed at the 95% confidence level with the R function [boot.ci] in the package *boot* [56, 57]. We used the R package *vioplot* [58] to construct violin plots of the relative abundances of the most abundant bacterial genera. Each NP specimen was assigned to a “bacterial profile group” based on the most abundant bacterial genus detected from the specimen. We generated alluvial plots of NP bacterial trajectories using the package *plotly* in Python [59].

We investigated associations between covariates and alpha diversity, and covariates and bacterial taxa, across three specimen collection intervals [interval A: 1 to 3 months (M01-M03), interval B: 4 to 6 months (M04-M06), and interval C: 7 to 12 months (M07-M12)] (Supplementary material, Section A). We elected to separately compare covariates across these intervals since microbiota profiles are strongly age-dependent over the first year of life and since not all participants had samples collected at each timepoint. For the analysis of association with covariates, we excluded participants where specimen collection was incomplete for the respective interval (A, B, or C). We performed differential abundance testing using Microbiome Multivariable Associations with Linear Models (MaAsLin2) [60] and Analysis of Composition of Microbiomes (ANCOM 2) [61]. We applied a random effects model to each of the three specimen collection intervals to account for repeated sampling.

Since statistical methods for beta diversity were not able to account for multiple sampling per participant (random effects could not be modelled), we investigated associations between covariates and beta diversity cross-sectionally at five timepoints in the first year (M01, M03, M06, M09, and M12) (Supplementary material, Section A). In addition, we used compositional tensor factorization (CTF) [62], which uses dimensionality reduction to incorporate information from participant-level patterns in microbiome composition across multiple samples, to identify associations between exposure variables and microbial composition across the full 12-month period. We visualized these differences over time using volatility control plots and used univariate linear models to explore associations between exposures and the three major ordination axes derived from CTF.

**Fig. 1:**
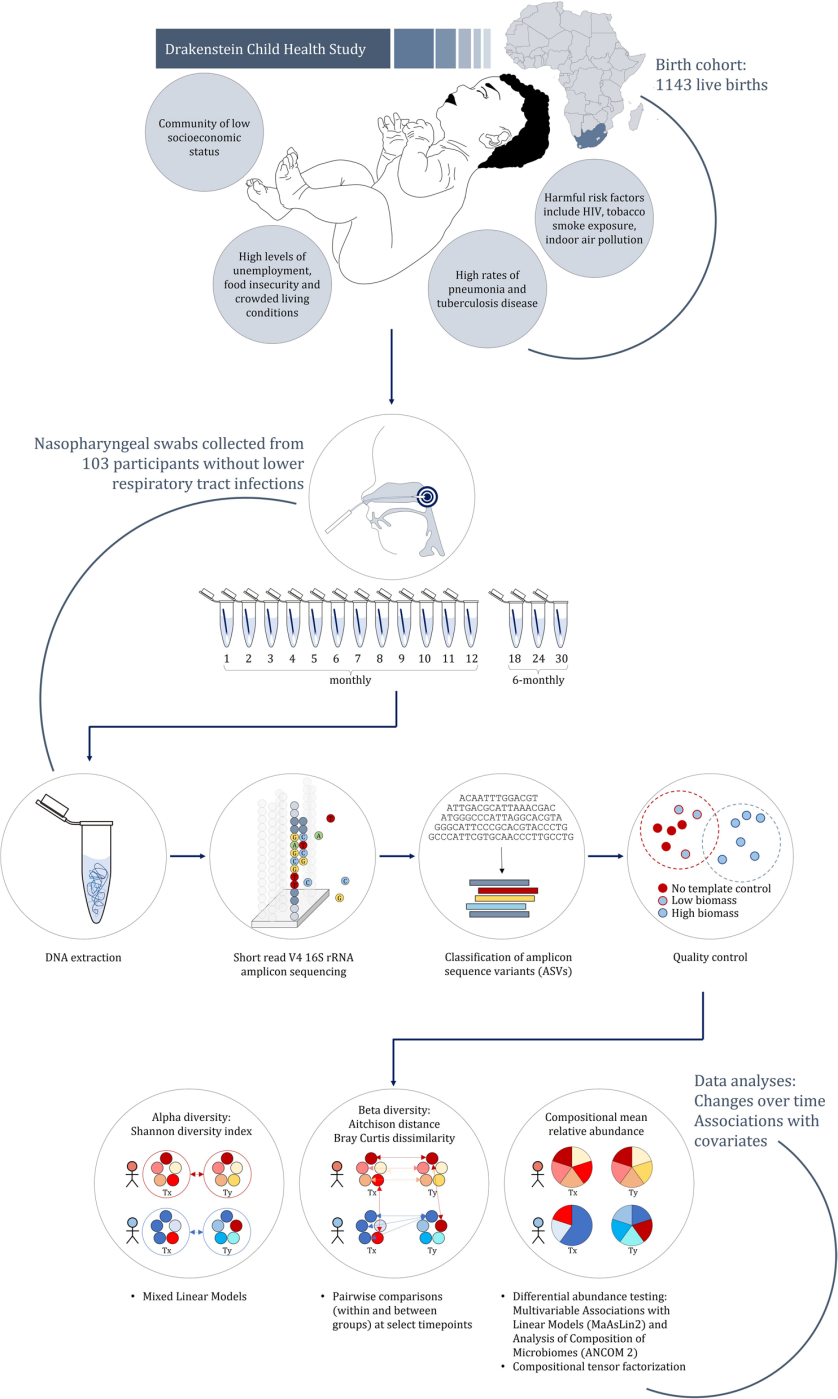
Summary of overall study design and analysis approach

Covariates tested included specimen collection season, sex, mode of delivery, gestational age, WAZ at birth, duration of exclusive breastfeeding, maternal smoking, pets in the home during the first 3 months of life, older siblings, day-care attendance, total monthly household income, maternal educational attainment, antibiotic administration, isoniazid (INH) prophylaxis, WAZ and height-for-age *z* score (HAZ) at 12 months, and HIV exposure. We categorized NP specimens as INH- and/or antibiotic-exposed if exposed < 100 days prior to collection (Supplementary Tables 3 and 4). The analysis code has been posted at https://github.com/yxia-code/hcm_analysis. The overall study design and analysis strategy is summarized in Fig. 1.

## Results

### Nasopharyngeal specimens, amplicon sequence variants, and participants

Bacterial profiles from the 24 mock community controls included in the five sequencing runs were reproducible and comparable to the theoretical compositions provided by the manufacturer (Supplementary material, Section B). We also observed high sequencing reproducibility from NP specimens randomly selected for repeat processing (Supplementary material, Section B).

Following bioinformatic processing and quality control processes, a total of 1358 NP specimens (and 1031 ASVs) from 103 participants were included to investigate changes in NP bacterial profiles over time. The median read count from NP specimens included was 20,900 (IQR: 16,719–26,036). The reasons for sample exclusion and ASV removal are detailed in Supplementary material Section B, Fig. S7.

To investigate associations between early life exposures and NP bacterial profiles during the first year of life, we excluded a further four participants due to incomplete specimen collection across intervals A, B, and C (Fig. S7). A total of 70/99 (71%), 85/99 (86%), or 77/99 (78%) participants had complete specimen collections across intervals A, B, or C, respectively.

### Participant characteristics

Characteristics of the participants are summarized in Table 1. Of the 103 children included in the study of longitudinal changes in the microbiota, most were female (59%) and were delivered by vaginal delivery (80%) and full term [median gestational age: 39 weeks (interquartile range (IQR): 38–40)] with normal weight-for-age *z* scores (WAZ) [median WAZ: − 0.28 (IQR: − 1.06–0.33)]). One quarter (24%) of infants were HIV-exposed but uninfected. Duration of exclusive breastfeeding was short, with 69% of participants exclusively breastfed for at least 1 month and only 20% for more than 4 months. Maternal smoking occurred in 15% of participants. Poor socioeconomic status was reflected by low total monthly income [less than 5000 ZAR (USD 320) per month for 87% of households] and low maternal educational attainment (69% of mothers had primary level education only).

**Table 1:** Participant characteristics

	**Study of NP microbiota over time**	**Study of association between NP microbiota and exposures**
	***N* = 103**	***N* = 99**
**Participant characteristics**	***n* (%)**	***n* (%)**
**Perinatal factors**		
Birth season		
Autumn (April–May)	10 (10)	10 (10)
Winter (June–August)	29 (28)	29 (29)
Spring (September–November)	26 (25)	23 (23)
Summer (December–March)	38 (37)	37 (38)
Sex		
Female	61 (59)	59 (60)
Mode of delivery		
Cesarean-section delivery	21 (20)	21 (21)
Gestational age		
Preterm (< 37 weeks)	14 (13)	13 (13)
37 to 40 weeks	76 (74)	74 (75)
> 40 weeks	13 (13)	12 (12)
WAZ at birth		
Low WAZ (< -1 SD below the mean WAZ score)	29 (28)	29 (29)
Medium WAZ (-1 to 1 SD of the mean WAZ score)	65 (63)	61 (62)
High WAZ (> 1 SD above the mean WAZ score)	10 (9)	9 (9)
Duration of exclusive breastfeeding		
< 1 month or none	32 (31)	30 (31)
Until 1 month	29 (28)	28 (28)
Until 2–3 months	21 (20)	21 (21)
Until 4–6 months	21 (20)	20 (20)
**Environmental exposures**		
Maternal tobacco use (self-reported)		
Yes	15 (15)	15 (15)
Pets in the home during the 1st 3 months of life		
Yes	23 (22)	23 (23)
Older siblings		
Yes	70 (68)	66 (67)
First day-care attendance (prior to specimen collection)		
At 0–6 months of age (yes)	7 (7)	6 (6)
At 7–12 months of age (yes)	12 (12)	12 (12)
**Sociodemographic variables**		
Total household income		
< 1000 ZAR (< 64 USD) per month	48 (47)	46 (47)
1000–5000 ZAR (64–320 USD) per month	42 (41)	41 (41)
> 5000 ZAR (> 320 USD) per month	13 (12)	12 (12)
Maternal educational attainment		
Completed primary level education	71 (69)	68 (69)
Completed secondary level education	32 (31)	31 (31)
**Infant exposures**		
Immunization cover		
Bacille Calmette-Guérin at birth	103 (100)	99 (100)
Diphtheria, tetanus, and acellular pertussis: 3 doses	103 (100)	99 (100)
Pneumococcal conjugate vaccine: 3 doses	102 (99)	98 (99)
Measles, mumps, rubella at 9 months	102 (99)	98 (99)
Oral polio vaccine: 3 doses	103 (100)	99 (100)
Antibiotic administration		
At 0–6 months of age (yes)	15 (15)	15 (15)
At 7–12 months of age (yes)	29 (28)	29 (29)
INH prophylaxis		
At 6–12 months of age (yes)	9 (9)	9 (9)
WAZ at 12 months of age		
Low WAZ (≤ 1 SD below the mean WAZ score)	17 (17)	17 (17)
Medium WAZ (− 1 to 1 SD of the mean WAZ score)	68 (66)	66 (67)
High WAZ (> 1 SD above the mean WAZ score)	18 (17)	16 (16)
HAZ at 12 months of age		
Low HAZ (≤ 1 SD below the mean HAZ score)	31 (30)	31 (31)
Medium HAZ (− 1 to 1 SD of the mean HAZ score)	64 (62)	61 (62)
High HAZ (> 1 SD above the mean HAZ score)	8 (8)	7 (7)
HIV-exposure		
HIV-exposed, uninfected	26 (25)	23 (23)

*HAZ* Height-for-age *z* score, *HIV* Human immunodeficiency virus, *TB-INH* Tuberculosis isoniazid, *SD* Standard deviation, *USD* US dollar, *WAZ* Weight-for-age *z* score, *ZAR* South African Rand

There was high immunization coverage for all expanded program on immunization vaccines (Table 1). Ten participants had a positive tuberculin skin test over the first year of life, nine of whom received INH prophylaxis. Hospitalization or antibiotic administration (for reasons other than LRTI) was recorded for 15% or 43% of infants, respectively.

### Dynamics of NP bacterial profiles during the first 30 months of life

**Fig. 2:**
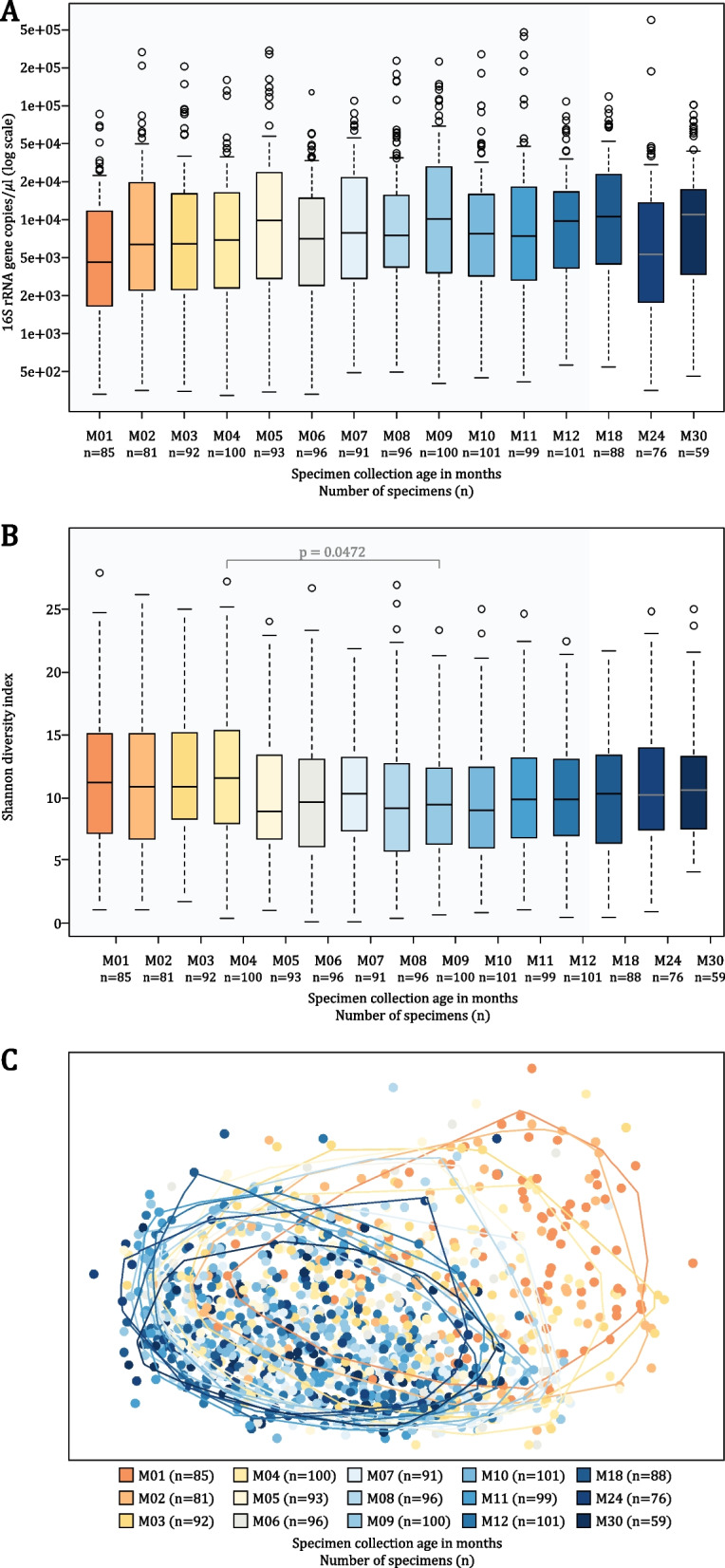
Bacterial density and diversity measured during the first 30 months of life. **A** Bacterial density (16S rRNA gene copies/μl) compared across timepoints. **B** Within-specimen bacterial diversity (Shannon diversity) compared across timepoints. One-way analysis of variance (ANOVA) was used to compare alpha diversity indices between timepoints. Tukey’s Honest Significant Difference test was implemented to compare each pair of timepoints simultaneously pairwise. Median values are presented by horizontal lines within each of the boxplots while upper and lower ranges of the boxplots represent the 75% and 25% quartiles, respectively. Maximum and minimum values, excluding outliers, are presented by whiskers. **C** Principal coordinate analysis of between-specimen bacterial diversity (Aitchison distance). Alpha bags (90%) are used to enclose observations from each of the timepoints, excluding the 10% of the observations at the extremes of each cluster. Specimen collection age (in months) and the number of specimens included at each timepoint are shown at the bottom of each panel

Bacterial density (16S rRNA gene copies/μl) was lowest at 1 month of life, but there were no significant differences through 30 months (Fig. 2A). Within-specimen bacterial diversity (Shannon diversity) measured at each timepoint showed an overall decrease after 4 months of life; however, no statistically significant differences were observed between consecutive timepoints throughout the study period (Fig. 2B). Changes in between-specimen bacterial diversity (Aitchison distance) with age are shown in Fig. 2C.

We used CTF to summarize the microbial trajectory of each child over 12 months. Each child’s time series was then represented as a single point on a compositional biplot, using the top two ordination axes, and showing the feature loadings (Fig. S8). Trajectory analysis (Fig. S9) for Axis 1 showed significant variation between participants at early timepoints, with convergence and decrease along this axis over time. Axis 1 was driven along the positive axis by ASV_ 30 (*Fusobacterium* spp.), ASV_20 (*Neisseria* spp.), and ASV_8 (*Corynebacterium* spp.), and along the negative axis by ASV_1 (*Moraxella* spp.), ASV_2, and ASV_3 (both *Haemophilus* spp.). In contrast, Axis 2 showed an increase with age, with convergence at 3 months, and was driven along the positive axis by ASV_ 24 (*Streptococcus* spp.), ASV_4 (*Corynebacterium* spp.), and ASV_20 (*Neisseria* spp.), and along the negative axis by ASV_8 (*Corynebacterium* spp.) and ASV_ 30 (*Fusobacterium* spp.).

**Fig. 3:**
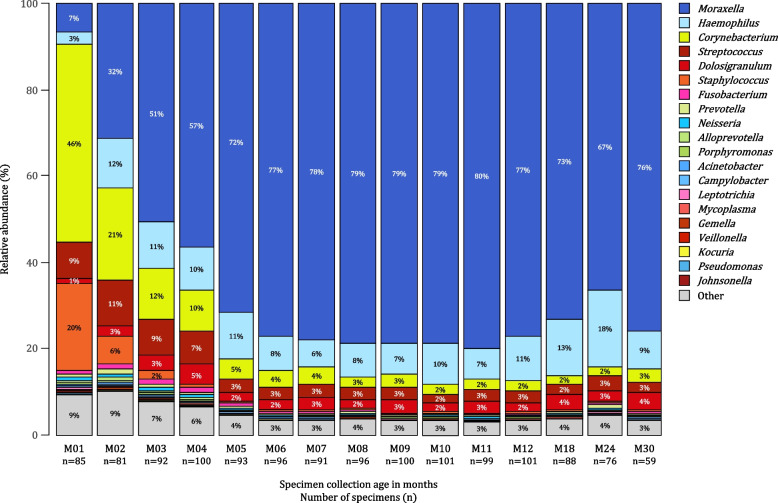
Barplots of compositional mean relative abundances of the 20 most abundant nasopharyngeal (NP) bacterial genera detected in 103 participants during the first 30 months of life. Each barplot represents compositional mean relative abundances for each of the 20 most abundant NP bacterial genera at each of the timepoints. The 20 most abundant bacterial genera were identified as the 20 bacterial genera with the highest sum of mean relative abundances across the first 30 months of life. Specimen collection age (in months) and the number of specimens analyzed at each of the timepoints are shown on the *X*-axis. Shades of colors are used to present phylum-level classification [shades of blue: Proteobacteria (and Campilobacterota, previously Epsilonproteobacteria), shades of yellow: Actinobacteria, shades of red: Firmicutes, shades of pink: Fusobacteriota, shades of green: Bacteroidetes]

Nasopharyngeal bacterial profiles during the first 30 months of life were dominated by six bacterial genera, including *Moraxella*, *Haemophilus*, *Corynebacterium*, *Streptococcus*, *Dolosigranulum*, and *Staphylococcus* (Fig. 3).

*Staphylococcus* and *Corynebacterium* were detected at highest compositional mean relative abundances at 1 month of life, with a tenfold decrease observed by 3 and 5 months of life for *Staphylococcus* and *Corynebacterium*, respectively (Fig. 3). *Corynebacterium* was a dominant colonizer in 32% (27/85), 21% (17/81), 13% (12/92), and 11% (11/100) of NP bacterial profiles at 1 to 4 months of life. *Staphylococcus* dominated 21% (18/85), 12% (10/81), 5% (5/92), and 6% (6/100) of NP bacterial profiles at 1 to 4 months of life.

**Fig. 4:**
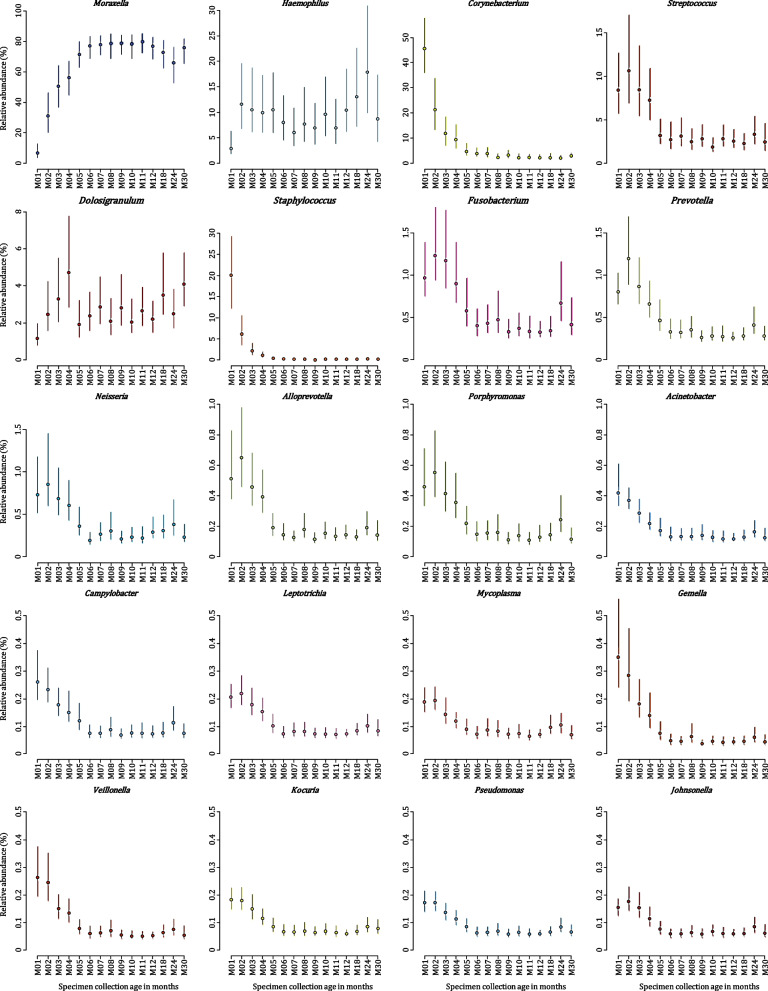
Bootstrap confidence intervals of estimates of compositional mean relative abundances of the 20 most abundant nasopharyngeal (NP) bacterial genera detected in 103 participants during the first 30 months of life. Bootstrap confidence intervals were computed at the 95% confidence level (vertical lines) for each of the 20 most abundant NP bacterial genera at each of the timepoints under study. Compositional mean relative abundances at each of the timepoints are presented by the dot on each bootstrap confidence interval line. Specimen collection age (in months) is shown on the *X*-axis. Shades of colors are used to present phylum-level classification [shades of blue: Proteobacteria (and Campilobacterota, previously Epsilonproteobacteria), shades of yellow: Actinobacteria, shades of red: Firmicutes, shades of pink: Fusobacteriota, shades of green: Bacteroidetes]. Note that the *Y*-axis scale varies

Compositional mean relative abundance of *Moraxella* increased fivefold by 2 months of age and 12-fold by 6 months whereafter it remained relatively constant (Figs. 3 and 4). *Moraxella* had highest relative abundance among 18% (15/85), 35% (28/81), 41% (38/92), and 55% (55/100) of NP bacterial profiles at 1 to 4 months of life, respectively. *Haemophilus* compositional mean relative abundance plateaued around two months of age. At 1 to 4 months of life, *Haemophilus* dominated 13% (11/85), 22% (18/81), 28% (26/92), and 19% (19/100) of NP bacterial profiles, respectively.

*Moraxella* and *Haemophilus* were detected at higher compositional mean relative abundances than any other bacterial genera at each of the timepoints after 4 months of age. However, the distributions of relative abundances for these two genera across children were different. *Moraxella* represented approximately 77% of the compositional mean relative abundances at each timepoint after 4 months and was the dominant genus in a similar proportion of specimens (60–70%) at each of these timepoints. In contrast, although *Haemophilus* only accounted for approximately 10% of the compositional mean relative abundances at each timepoint, it was the dominant genus in 20–30% of specimens at each timepoint after 4 months.

*Streptococcus* compositional mean relative abundance decreased threefold by 4 months; whereafter, it remained relatively constant (Figs. 3 and 4). *Streptococcus* was most abundant among 8% (7/85), 4% (3/81), 8% (7/92), and 2% (2/100) of NP bacterial profiles at 1 to 4 months of life, respectively. *Streptococcus* was dominated by two ASVs (ASV_7 and ASV_10). Mean relative abundance of ASV_10 decreased from 1.7% at 1 month of life to < 0.1% at 4 months, whilst mean relative abundances of ASV_7 remained relatively stable, between 0.3% and 1.4% across all timepoints.

Most (99%) NP specimens were dominated (sum of relative abundances ≥ 90%) by 10 or fewer bacterial genera, with 93% NP specimens dominated by 5 or fewer bacterial genera (Supplementary Fig. S10).

**Fig. 5:**
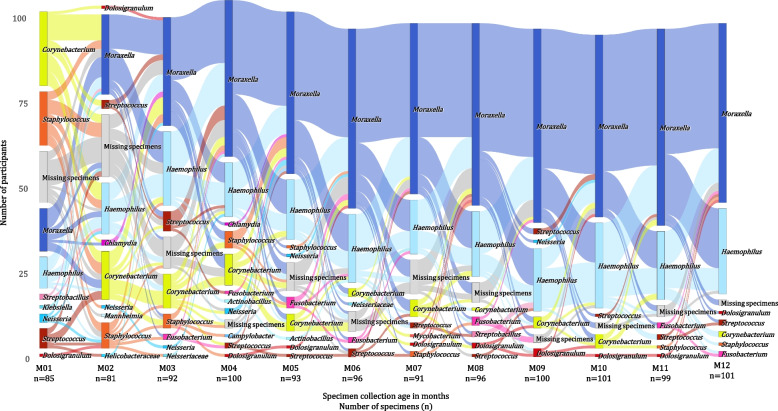
Alluvial plot showing changes in nasopharyngeal (NP) bacterial profiles among the 103 participants based on the most abundant bacterial genus detected at each preceding timepoint during the first year of life. Vertical bars at each timepoint represent the number of participants from which respective bacterial genera (shades of yellow: Actinobacteria; shades of red: Firmicutes, shades of blue: Proteobacteria, shades of pink: Fusobacteriota) were identified as most abundant. The proportion of participants with missing specimens at each timepoint is shown in gray. Specimen collection age (in months) and the number of specimens analyzed at each of the timepoints are denoted on the *X*-axis. Each participant is represented by a ribbon moving along the *X*-axis according to specimen collection age and *Y*-axis according to the most abundant bacterial genus detected at each of the timepoints. The color assigned to each participant ribbon is based on the most abundant bacterial genus detected at each preceding timepoint, highlighting sequential changes

Following participants over time, we observed substantial instability of profiles during the first 4 months of life (Fig. 5). We observed more stable profiles after 4 months of life with most shifts in profiles occurring between *Moraxella*- and *Haemophilus*-dominated profiles. Profiles dominated by *Streptococcus*, *Staphylococcus*, *Corynebacterium*, and *Neisseria* diminished over time.

### Determinants of infant nasopharyngeal bacterial profiles

Associations between covariates and alpha diversity, beta diversity, and relative abundances of bacterial taxa (genus and ASV level) were investigated across three specimen collection intervals [interval A: one to three months (M01-M03), interval B: 4 to 6 months (M04-M06), and interval C: 7 to 12 months (M07-M12)]. Differential abundance testing was performed on 54, 44, and 47 bacterial genera and 95, 76, and 74 ASVs across intervals A, B, and C, respectively (Supplementary Table S5).

### Association between season of specimen collection and bacterial profiles

Apart from age, season of specimen collection was the exposure most consistently associated with microbiota profiles. Specimens collected during summer yielded higher within-specimen diversity compared to most other collection seasons (Table 2; Fig. S11A). Similarly, specimens collected during summer had the highest between-specimen diversity across most timepoints (Aitchison distance and Bray-Curtis dissimilarity) while specimens collected during autumn had the lowest between-specimen diversity across four of the five timepoints studied (Figs. S12A and S13A).

**Table 2:** Early life exposures associated with nasopharyngeal bacterial alpha (Shannon) diversity

Covariate	Interval A: M01-M03	*P* value	Interval B: M04-M06	*P* value	Interval C: M07-M12	*P* value
Specimen collection season	**Summer > Autumn**	0.008**	Summer > Autumn	0.315	Summer > Autumn	0.150
	Summer > Winter	0.261	Summer > Winter	0.925	**Summer > Winter**	0.083*
	Summer > Spring	0.415	Summer < Spring	0.358	Summer > Spring	0.570
	Summer > Autumn	0.268	**Summer > Autumn**	0.005**	**Summer > Autumn**	0.071*
	Summer < Winter	0.388	**Summer > Winter**	0.009**	**Summer > Winter**	< 0.001**
	Summer < Spring	0.525	Summer > Spring	0.891	**Summer > Spring**	0.004**
Gestational age	More than 40 weeks > Less than 37 weeks	0.903	**More than 40 weeks < Less than 37 weeks**	0.057*	More than 40 weeks < Less than 37 weeks	0.241
	More than 40 weeks < 37–40 weeks	0.439	**More than 40 weeks < 37–40 weeks**	0.093*	More than 40 weeks < 37–40 weeks	0.372
	More than 40 weeks > Less than 37 weeks	0.659	**More than 40 weeks < Less than 37 weeks**	0.097*	More than 40 weeks < Less than 37 weeks	0.211
	More than 40 weeks < 37–40 weeks	0.339	More than 40 weeks < 37–40 weeks	0.254	More than 40 weeks < 37–40 weeks	0.114
Duration of exclusive breastfeeding	None/less than 1 month < Until 1 month	0.437	None/less than 1 month < Until 1 month	0.789	None/less than 1 month < Until 1 month	0.599
	None/less than 1 month < Until 2 months	0.905	None/less than 1 month < Until 2 months	0.671	None/less than 1 month < Until 2 months	0.325
	None/less than 1 month < Until 3 months	0.426	None/less than 1 month > Until 3 months	0.182	None/less than 1 month < Until 3 months	0.658
			**None/less than 1 month < Until 4 months**	< 0.001**	**None/less than 1 month < Until 4 months**	0.049**
			None/less than 1 month > Until 5 months	0.905	None/less than 1 month > Until 5 months	0.762
			None/less than 1 month > Until 6 months	0.581	None/less than 1 month < Until 6 months	0.499
	None/less than 1 month < Until 1 month	0.740	None/less than 1 month > Until 1 month	0.691	**None/less than 1 month < Until 1 month**	0.004**
	None/less than 1 month < Until 2 months	0.697	None/less than 1 month > Until 2 months	0.927	**None/less than 1 month < Until 2 months**	0.006**
	None/less than 1 month < Until 3 months	0.912	None/less than 1 month > Until 3 months	0.809	None/less than 1 month < Until 3 months	0.358
			**None/less than 1 month < Until 4 months**	0.008**	**None/less than 1 month < Until 4 months**	0.050*
			None/less than 1 month > Until 5 months	0.918	None/less than 1 month < Until 5 months	0.815
			None/less than 1 month > Until 6 months	0.279	None/less than 1 month < Until 6 months	0.425
Maternal smoking	Yes > No	0.350	Yes > No	0.317	Yes > No	0.420
	**Yes > No**	0.089*	Yes > No	0.113	Yes > No	0.209
Pets in the home during the 1st 3 months of life	Yes < No	0.116	Yes > No	0.651	**Yes > No**	0.095*
	Yes < No	0.491	Yes > No	0.279	**Yes > No**	0.084*
Older siblings	Yes > No	0.405	Yes > No	0.764	**Yes > No**	0.090*
	Yes > No	0.600	**Yes > No**	0.030**	Yes > No	0.362
Maternal educational attainment	Primary level education < Secondary level education	0.936	**Primary level education < Secondary level education**	0.003**	Primary level education < Secondary level education	0.779
	Primary level education < Secondary level education	0.918	Primary level education < Secondary level education	0.198	Primary level education < Secondary level education	0.346
Antibiotics prior to specimen collection	Not tested	-	Yes > No	0.991	Yes > No	0.239
	Not tested	-	Yes < No	0.623	**Yes > No**	0.077*
HIV-exposure	HIV-unexposed > HIV-exposed, uninfected	0.560	**HIV-unexposed > HIV-exposed, uninfected**	0.050*	HIV-unexposed > HIV-exposed, uninfected	0.341
	HIV-unexposed < HIV-exposed, uninfected	0.423	HIV-unexposed > HIV-exposed, uninfected	0.281	HIV-unexposed > HIV-exposed, uninfected	0.071*
Height-for-age z-score (HAZ) at 12 months	**High**^**a**^** HAZ scores < Low**^**b**^** HAZ scores**	0.026**	High^a^ HAZ scores < Low^b^ HAZ scores	0.381	High^a^ HAZ scores < Low^b^ HAZ scores	0.202
	**High**^**a**^** HAZ scores < Medium**^**c**^** HAZ scores**	0.009**	High^a^ HAZ scores < Medium^c^ HAZ scores	0.694	High^a^ HAZ scores < Medium^c^ HAZ scores	0.250
	**High**^**a**^** HAZ scores < Low**^**b**^** HAZ scores**	0.068*	High^a^ HAZ scores > Low^b^ HAZ scores	0.851	High^a^ HAZ scores < Low^b^ HAZ scores	0.461
	**High**^**a**^** HAZ scores < Medium**^**c**^** HAZ scores**	0.058*	High^a^ HAZ scores > Medium^c^ HAZ scores	0.505	High^a^ HAZ scores < Medium^c^ HAZ scores	0.689

Within-specimen (alpha) diversity *p* values were adjusted for specimen collection age and season

*HIV* Human immunodeficiency virus, *TB-INH* Tuberculosis isoniazid

^*^p values < 0.10; ^**^p values < 0.05

^a^High HAZ: > 1 standard deviation above the mean HAZ score

^b^Low HAZ: ≤ 1 standard deviation below the mean HAZ score

^c^Medium HAZ score: − 1 to 1 standard deviation of the mean HAZ score

**Fig. 6:**
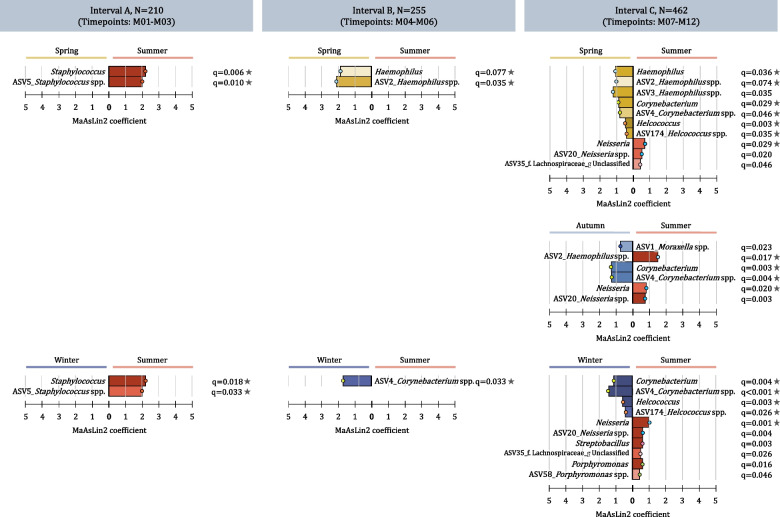
Differential abundance testing for specimen collection season and perinatal factors. Differential abundance testing results are presented for specimen collection season across three specimen collection intervals (interval A: M01-M03, interval B: M04-M06, and interval C: M07-M12). Differentially abundant taxa are shown at genus- and ASV-level. Taxa with *q* values < 0.10 were deemed differentially abundant using Microbiome Multivariable Associations with Linear Models (MaAsLin2). The color intensity of each taxon bar represents the level of significance (darker shades represent smaller *q* values). The horizontal length of each taxon bar shows the MaAsLin2 coefficient. Stars next to *q* values show differentially abundant taxa as per Analysis of Composition of Microbiomes (ANCOM 2) (W statistic > 0.6)

We detected higher relative abundances of *Staphylococcus* from specimens collected in summer compared to spring and winter (interval A) (Fig. 6). We found higher relative abundances of *Haemophilus* from specimens collected in spring compared to summer (intervals B and C). We observed higher relative abundances of *Corynebacterium* from specimens collected in winter compared to summer (interval B and C) and autumn compared to summer (interval C). *Streptococcus*, *Neisseria*, *Fusobacterium*, *Streptobacillus*, *unclassified ASV_35* (family Lachnospiraceae), *Porphyromonas*, *Alloprevotella*, and *Gemella* were detected at higher relative abundances from specimens collected during summer compared to other seasons (interval C).

### Association between perinatal factors and bacterial profiles

**Fig. 7:**
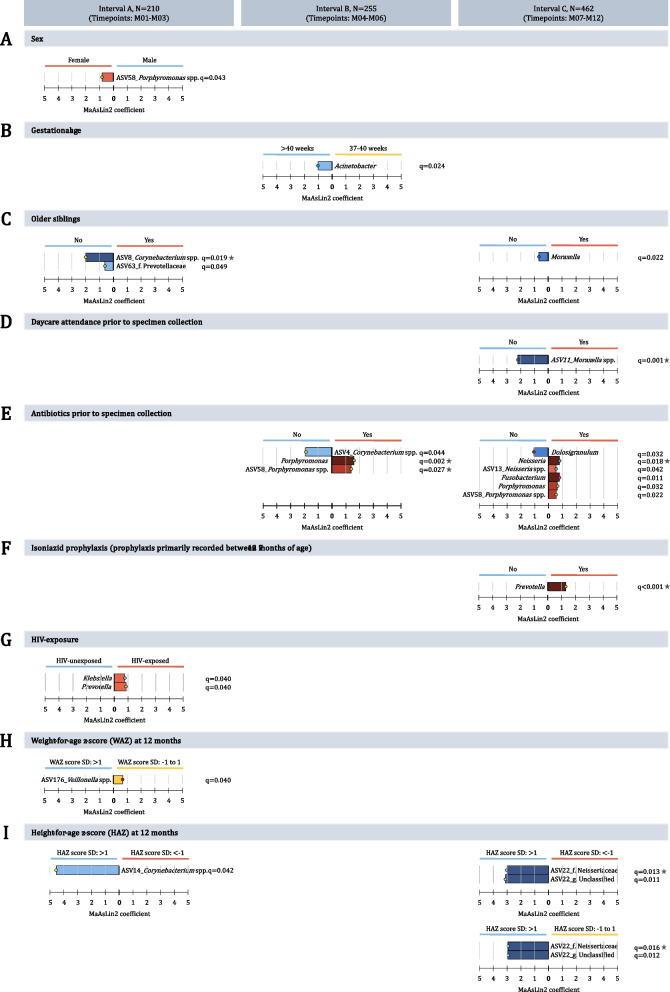
Differential abundance testing for environmental or sociodemographic variables. Differential abundance testing results are presented for **A** sex, **B** gestational age, **C** older siblings, **D** daycare attendance (prior to specimen collection), **E** first report of antibiotic administration (prior to specimen collection), **F** tuberculosis isoniazid prophylaxis, **G** HIV exposure, **H** weight-for-age *z* score measured at 12 months, and **I** height-for-age *z* score measured at 12 months across three specimen collection intervals (interval A: M01-M03, interval B: M04-M06, and interval C: M07-M12). Differentially abundant taxa are shown at genus- and ASV-level. Taxa with *q* values < 0.10 were deemed differentially abundant using Microbiome Multivariable Associations with Linear Models (MaAsLin2). The color intensity of each taxon bar represents the level of significance (darker shades represent smaller *q* values). The horizontal length of each taxon bar shows the MaAsLin2 coefficient. Stars next to *q* values show differentially abundant taxa as per Analysis of Composition of Microbiomes (ANCOM 2) (W statistic > 0.6)

Sex, gestational age, and mode of delivery were not consistently associated with diversity or relative abundance of taxa (Fig. 7, S11B-D). Between-specimen diversity (Bray–Curtis dissimilarity) was higher among infants born < 37 weeks of gestation compared to infants born > 40 weeks of gestation, from 3 months of age onwards (Fig. S13D). Our ability to detect associations with exclusive breastfeeding was limited by the overall short duration of exclusive breastfeeding; however, we did note that very short duration of exclusive breastfeeding (< 1 month) was associated with lower within-specimen diversity across several time periods (Table 2; Fig. S11F).

### Association between environmental or sociodemographic exposures and bacterial profiles

At 6 months of age, between-specimen diversity (Bray–Curtis dissimilarity) was higher between specimens from infants whose mothers smoked when compared to specimens from infants whose mothers did not (Fig. S16A), with a similar trend observed at nine months of age.

Infants without older siblings had higher relative abundances of ASV_8 (*Corynebacterium* spp.) across interval A and *Moraxella* in interval C, compared to infants with older siblings (Fig. 7C).

We identified consistently higher (but individually non-significant) within-specimen diversity among infants not attending day-care compared to infants attending day-care (Fig. S14D). We also observed higher between-specimen diversity (Bray Curtis dissimilarity) among infants not attending day-care (Fig. S16D). Relative abundance of *Moraxella* ASV_11 was higher across interval C for infants not attending day-care (Fig. 7D).

Infants with no antibiotic exposure in the three months prior to specimen collection had higher relative abundances of *Corynebacterium* (interval B) and *Dolosigranulum* (interval C) (Fig. 7E), compared to infants who had received antibiotics. Conversely, infants who received antibiotics prior to specimen collection had higher *Porphyromonas* (intervals B and C) and *Neisseria* and *Fusobacterium* (interval C). Infants who received INH prophylaxis had higher relative abundances of *Prevotella* at interval C (Fig. 7F).

HIV-unexposed infants had a trend to consistently higher (but individually non-significant) within-specimen diversity when compared to HIV-exposed infants (Table 2; Fig. S17C). At interval A, HIV-exposed infants had higher relative abundances of *Klebsiella* and *Prevotella* (Fig. 7G).

Infants with lower HAZ scores measured at 12 months of age had higher within-specimen diversity compared to infants with higher HAZ scores (Table 2, Fig. S17E). At 6, 9, and 12 months of age infants with low HAZ scores (measured at 12 months of age) had higher between-specimen diversity (Bray–Curtis dissimilarity) compared to infants with higher HAZ scores (Figs. S18E and S19E). High HAZ scores at 12 months of age were associated with higher relative abundances of ASV_14 (*Corynebacterium*) over interval A, and ASV_22 (family Neisseriaceae) over interval C (Fig. 7I).

In addition to analyzing associations between exposures separately across each of the three time intervals, we explored associations between exposures and the overall microbial trajectory of each child, summarized using CTF (Fig. S9). Axis 1 was significantly negatively associated with low gestational age (*p* < 0.001) and HIV-exposure (*p* = 0.05) and positively associated with low income (*p* < 0.001), pets in the household (*p* = 0.012) and low or middle (vs. high) WAZ score at birth (*p* < 0.001). Axis 2 was significantly negatively associated with vaginal delivery (*p* < 0.001) and HIV-exposure (*p* = 0.05) and middle (vs high) income (*p* = 0.016).

## Discussion

Our study investigating NP bacterial profiles in an intensively sampled and well-phenotyped South African birth cohort showed similarities in NP bacterial trajectories over time when compared to most previous reports from high-income countries. Dominant colonizers during the first 2 months of life included *Corynebacterium* and *Staphylococcus*, whilst *Moraxella* dominated profiles at 6 months [4, 7, 8, 12, 16, 21, 63]. A striking difference in NP bacterial trajectories observed from our cohort, compared to previous reports, was the rapid loss of *Corynebacterium* and *Dolosigranulum* with early transition to profiles dominated by *Moraxella* and *Haemophilus* [4, 7, 8, 12, 16, 21, 63]. Studies from high-income countries generally reported higher mean relative abundances for *Corynebacterium*, *Dolosigranulum*, *Streptococcus*, and *Staphylococcus* at 6 months of age when compared to our study, with mean relative abundances of *Moraxella* below 50% [4, 8, 12, 16, 21]. These findings may have implications for child health, since *Moraxella*, *Haemophilus*, and *Streptococcus* have been associated with LRTI in childhood, whilst *Corynebacterium* and *Dolosigranulum* are considered health-associated taxa in early life [4, 8, 20, 64, 65]. Indeed, infants in the broader DCHS cohort have a very high incidence of LRTI during the first year of life [66, 67].

Early colonization with a *Moraxella*- or *Dolosigranulum/Corynebacterium-*dominated profile have been associated with more stable bacterial colonization patterns during the first 2 years of life [4]. Conversely, *Streptococcus*- and *Haemophilus*-dominated profiles early in life were marked by high levels of change and dispersion of profiles over time [4]. Early life NP bacterial profiles may therefore drive NP bacterial succession patterns which may have important implications for respiratory health [4]. In our study of healthy children, instability in NP bacterial profiles was highest throughout the first four months of life. Thereafter, more stable profiles (with shifts primarily occurring between *Moraxella*- and *Haemophilus*-dominated profiles) occurred. Results from our study highlight the importance of high frequency of sampling to understand the developmental dynamics of NP bacterial profiles.

In our study, mean relative abundances of bacterial genera identified at 12 months of age did not differ significantly from those at 6 months. Conversely, studies from Australia and the Netherlands have reported an increase in mean relative abundance of *Moraxella*,* Haemophilus*, and *Streptococcus* and a decrease in *Corynebacterium* when comparing profiles at 12 months to 6- or 9-month timepoints [4, 7, 8]. One other study from The Gambia showed higher relative abundance of *Streptococcus* in early life NP bacterial communities; however, children were primarily recruited from communities unvaccinated with pneumococcal conjugate vaccine [68].

To date, most studies investigating NP bacterial dynamics during infancy have been done in high-income countries [4, 7, 12, 16, 20, 63, 68–71] where sociodemographic conditions and environmental exposures may be very different compared to LMICs. These exposures could impact on the dynamics of NP bacterial profiles [17] and susceptibility to LRTI [29–31]. A striking finding from our study, using multiple analytical approaches, was that only a few of the broad range of exposures measured had a consistent impact on microbial profiles across time intervals, and the effect size of these exposures was relatively modest.

Specimen collection season was an important determinant of NP bacterial diversity [72], and relative abundance of several bacterial taxa. *Staphylococcus* (ASV_5) was detected at significantly higher relative abundance during summer in the first 3 months of life. Although short fragment 16S rRNA gene amplicon sequencing does not allow for species-level identification, previous culture and genotyping data on the acquisition of *Staphylococcus aureus* within the DCHS [73] suggests that ASV_5 may represent *S. aureus*. In support of these findings, a review evaluating seasonality in *S. aureus* colonization and infection of different body sites reported trends towards increased *S. aureus* carriage and infections during warmer weather [74]. However, a further study of NP specimens from 72 infants without upper respiratory tract infection (URTI), sampled during the first 6 months of life, showed a significantly higher prevalence of *S. epidermidis* during summer [75]. Across four to 12 months of life, *Haemophilus* relative abundance was highest during spring, consistent with findings from a previous study of infants in Perth, Australia [69], which is on the same latitude and has similar seasonal climate to Cape Town.

The effect of cesarean-section delivery on infant bacterial communities, particularly in the gastrointestinal tract, has been widely studied [76]. Unlike previous reports from high-income countries [7, 12], our study which used MaAsLin2 and the relatively conservative ANCOM 2 method for testing differential abundance [61] did not find statistically significant differences in relative abundances of genera previously associated with mode of delivery. However, there were relatively small numbers of children born by cesarean section in our study. Studies performed in the Netherlands reported limited direct impact of mode of delivery on NP bacterial profiles at the time of delivery, with differences emerging over subsequent months [12]. These findings suggest that changes in NP bacterial profiles among vaginal and cesarean-section delivered infants may be mediated by other early life exposures such as feeding practices [4, 7, 12] or antibiotic use [7].

Several studies have reported associations between early life feeding practices and NP bacterial communities [4, 7, 20, 63], yet our data did not show similar associations. However, duration of exclusive breastfeeding was very short in our cohort. A study conducted in the Netherlands reported that exclusively breastfed infants demonstrate higher relative abundances of *Corynebacterium* and *Dolosigranulum*, but reduced abundances of *Staphylococcus*, *Prevotella*, and *Veillonella* compared to formula-fed infants at 6 weeks of age [63].

Co-habiting with older siblings has been associated with lower abundances of *Staphylococcus* [69] and higher abundances of *Haemophilus* [69] (*H. influenzae* [77]), *Streptococcus* [69] (*S. pneumoniae* [77]), and *Moraxella* [69] during infancy. Our study suggests that exposure to older children may be a key factor associated with early loss of health-associated *Corynebacterium*; however, *Moraxella* relative abundance at 7–12 months of age was lower in children with older siblings. Exposure to older children may also occur during day-care attendance. We found that infants attending day-care had lower relative abundance of ASV_11 (*Moraxella spp.*). *Moraxella* has been previously reported at higher relative abundances from infants attending day-care [69]; however, ASV_11 was not the dominant *Moraxella* sequence variant in our study.

Prior antibiotic therapy was clearly associated with changes in the microbiota. Relative abundances of health-associated *Corynebacterium* and *Dolosigranulum* were reduced in infants with recent antibiotic exposure, as previously reported [7, 16, 69]. In addition, we found that gram-negative anaerobes (*Porphyromonas*, *Fusobacterium*, and *Prevotella*) had higher relative abundances in infants exposed to antibiotics, including INH, used for tuberculosis preventative therapy. Nasopharyngeal enrichment of oral anaerobes has been reported from hospitalized children with invasive pneumococcal disease following antibiotic treatment [78]. Longitudinal studies should investigate whether repopulation of the nasopharynx following antibiotic exposure results from expansion of oral anaerobic bacteria and whether such repopulation could be manipulated through oral hygiene and pre- or probiotics [78].

Despite associations between HIV and LRTI, few studies have compared NP bacterial community composition in HIV-exposed and HIV-unexposed infants. A study performed in Botswana reported lower relative abundances of *Dolosigranulum* from HIV-infected children but higher relative abundances of *Klebsiella* from HIV-exposed children [79]. In support of this, we found that *Klebsiella* and *Prevotella* ASVs had higher relative abundance among HIV-exposed compared to HIV-unexposed infants, but only across the first 3 months of life. Studies of lower airway microbial communities have previously reported enrichment of select oral commensal bacteria including *Veillonella*, *Prevotella*, and *Streptococcus* among HIV-infected patients [80]. Enrichment of these oral commensals has been associated with pneumonia, pulmonary tuberculosis, and chronic obstructive pulmonary disease [80].

Longitudinal modeling of associations between the microbiota and covariates is complex, particularly in early life, when rapid changes in composition occur. We used CTF to reduce each child’s microbial profile over the time series to a point in three-dimensional space, and then modeled the association between each ordination axis and exposure variables. This analysis revealed several additional potential associations, for example between HIV-exposure or low gestational age and a microbial trajectory driven by *Moraxella* and *Haemophilus* genera. However, interpretation of these associations is not straightforward, and further validation of these findings is needed.

The strengths of our study include intensive sampling and careful and detailed phenotyping of participants who experienced a range of relevant and previously understudied exposures. We identified relatively few consistent associations between exposures and microbial profiles. This may be because the ability to track associations over time allows us to exclude spurious findings observed at a single timepoint. Our study has several limitations. The use of short fragment 16S rRNA gene amplicon sequencing precluded species-level taxonomic assignment and does not provide information on absolute abundance of bacteria. The sample size was relatively modest, which may have limited our ability to detect subtle associations; however, there were few instances in which consistent, but non-significant, effects were observed over time, suggesting that increased sample size would not substantially alter our findings. Direct comparisons of NP bacterial profiles across studies may be complicated by several factors. These include 16S rRNA gene amplicon library preparation protocols [81–83], bioinformatic pipelines [84, 85], in silico quality control approaches [38], and the manner in which authors report their findings (for example, arithmetic versus compositional means of relative abundances of bacterial taxa or assignment of specimen clusters using varying definitions). We did not address mechanistic relationships between bacterial taxa and exposures.

In conclusion, our study is the first to longitudinally investigate NP bacterial dynamics and their determinants from healthy infants without LRTI residing in a in a low-resource setting with high LRTI incidence. NP bacterial profiles followed similar trajectories to those reported from high-income countries, but with rapid loss of health associated taxa *Corynebacterium* and *Dolosigranulum* and early replacement with *Moraxella* and *Haemophilus* [28]. Season and antibiotic exposure were key determinants of NP bacterial profiles early in life; however, succession of profiles appeared to be robust to a broad range of other exposures. Stochastic events, such as acquisition of a new taxon, and microbial interactions, should be further explored as important determinants of microbial succession in this niche.

## Supplementary Information

**Additional file 1: Section A.** Extended methods (Figure S1). **Section B.** Extended results - sequencing controls and sample selection (Figures S2-S7). **Section C.** Extended results - Figures S8-S19. **Section D.** Extended references. **Table S1.** RDP classifier implementation for DADA2 and SILVA version: ASV table. **Table S2.** RDP classifier implementation for DADA2 and SILVA version: taxonomic classification. **Table S3.** Metadata file: NP specimens. **Table S4.** Metadata file: Participants. **Table S5.** Differential abundance testing.

## Data Availability

Sequence data and subject characteristics are available in the National Center for Biotechnology Information (NCBI) Sequence Read Archive (SRA) under the BioProject ID PRJNA790843 and PRJNA548658. The ASV table, taxonomic classification, sample metadata, and participant metadata are available in Supplementary Tables 1–4.
