# Supplementary material for: Succession and determinants of the early life nasopharyngeal microbiota in a South African birth cohort
Source: Microbiome. 2023 Jun 5;11:127. doi: 10.1186/s40168-023-01563-5 (PMC10240772; doi:10.1186/s40168-023-01563-5)
Supplement: Supplementary file 2 — Additional file 1: Section A. Extended methods (Figure S1). Section B. Extended results - sequencing controls and sample selection (Figures S2-S7). Section C. Extended results - Figures S8-S19. Section D. Extended references. Table S1. RDP classifier implementation for DADA2 and SILVA version: ASV table. Table S2. RDP classifier implementation for DADA2 and SILVA version: taxonomic classification. Table S3. Metadata file: NP specimens. Table S4. Metadata file: Participants. Table S5. Differential abundance testing. [file 40168_2023_1563_MOESM1_ESM.zip › Healthy cohort_Supplementary_13 March_clean.docx]

Supplementary appendix for

**Determinants and succession of the early life nasopharyngeal bacteriome in a low-income community**

Shantelle Claassen-Weitz, Sugnet Gardner-Lubbe, Yao Xia, Kilaza S Mwaikono, Stephanie Harris Mounaud, William C. Nierman, Heather J. Zar, Mark P. Nicol*

*Correspondence to: mark.nicol@uwa.edu.au

This PDF file includes:

Section A. Extended methods (Figure S1)

Section B. Extended results - sequencing controls and sample selection (Figures S2-S7)

Section C. Extended results - Figures S8-S19, (Table S1 see separate spreadsheet)

Section D. Extended references

**Section A: Extended methods**

**Sequencing controls**

Each sequencing run consisted of four 96-well plates (384 reactions). A set of sequencing controls were included alongside NP specimens on each of the 96-well plates [1]. We included at least one mock community control [1-in-10 fold dilutions of bacterial mock community DNA controls “BEI-DNA” (HM-783D, BEI Resources, NIAID, NIH as part of the Human Microbiome Project, Manassas, VA, USA) and/or “Zymobiomics-DNA” (ZymoBIOMICS™ Microbial Community DNA Standard, catalogue no. D6305, Zymo Research Corp., Irvine, CA, USA)] per 96-well plate for validation of sequence quality control (Figure S1). We randomly selected at least one NP specimen per 96-well plate for repeat amplification and sequencing within each run (“within-run repeat”) and between different runs (“between-run repeat”) for assessing sequencing reproducibility (Figure S1). We included a minimum of one no template control (NTC) (neat PrimeStore^®^ Molecular Transport medium processed for nucleic acid extraction alongside NP specimens) to validate sequence quality from low biomass NP specimens and identify potential contaminant amplicon sequence variants (Figure S1).

**Figure S1** **Nasopharyngeal (NP) specimens and sequencing controls processed via 16S rRNA gene amplicon sequencing.**

A) Longitudinal collection of NP specimens: NP swabs (FLOQSwab^TM^, Copan Diagnostics, CA, USA) suspended in PrimeStore® Molecular Transport medium (Longhorn Vaccines & Diagnostics, MD, USA); B) Nucleic acid extraction: NP specimens and no template controls (NTCs); and C) 16S rRNA library preparation and sequencing: 1-in-10 fold dilutions of microbial mock community DNA controls [HM-783D (BEI Resources, NIAID, NIH as part of the Human Microbiome Project, VA, USA) (“BEI-DNA”) and/or ZymoBIOMICS™ Microbial Community DNA Standard (catalogue no. D6305, Zymo Research Corp., Irvine, CA, United States) (“Zymobiomics-DNA”)], nucleic acid extracts from NP specimens randomly selected for repeat amplification and sequencing (“within-run repeats”), nucleic acid extracts from NP specimens randomly selected from a previous sequencing run for repeat amplification and sequencing (“between-run repeats”), nucleic acid extracts from NP specimens and NTCs (neat PrimeStore® Molecular Transport medium processed for nucleic acid extraction alongside NP specimens).

**Amplicon library generation and sequencing**

We did two-step amplification of the V4 hypervariable region of the 16S rRNA gene using 7 µl of nucleic acid and 7 µl of amplicon as template, respectively [2]. We pooled purified short fragment 16S rRNA gene amplicons at equimolar concentration (70 ng) and determined the pooled library fragment size and concentration using the Agilent DNA 1000 Kit (Agilent Technologies, Santa Clara, CA, USA) and the KAPA Library Quantification Kit (catalogue no. KK4844, KAPA Biosystems, Boston, MA, USA), respectively. Library loading concentrations ranged between 5.5 pM and 7 pM per library across respective sequencing runs to obtain optimal flow cell loading concentrations. Library dilution and denaturing steps have been described elsewhere [2, 3]. Each library contained a 15% PhiX Control v3 spike-in (catalogue no. FC-110-3001, Illumina, San Diego, CA, USA). We spiked 3.4 µl of each of the 100 µM custom sequencing primers (Integrated DNA technologies, Coralville, IA, USA) into the MiSeq Reagent Kit v3 (600-cycle) Reagent Cartridge (Illumina, San Diego, CA, USA) [sequencing primer read 1: 5’-TATGGTAATTGTGTGCCAGCHGCYGCGGTAA-3’, sequencing primer read 2: 5’-AGTCAGTCAGCCGGACTACHVGGGTWTCTAAT-3’ and index sequence: 5’-ATTAGAWACCCBDGTAGTCCGGCTGACTGACT-3]. We sequenced the denatured libraries on the Illumina^®^ MiSeq^TM^ platform [4].

**Bioinformatic steps**

After assessing the quality of demultiplexed paired-end reads via FastQC [5] and MultiQC [6], we used the DADA2 pipeline [7] (wrapped in the Nextflow algorithm [8]) to filter and trim reads, infer amplicon sequence variants (ASVs), and assign taxonomy to ASVs. We trimmed and truncated forward reads at 24 and 248 bases (trimFor = 24; truncFor = 248) and reverse reads at 25 and 235 bases (trimRev = 25; truncRev = 235). We also truncated reads at the first instance of a quality score <2 (truncQ = 2), trimmed reads containing >2 expected errors (maxEEFor = 2; maxEERev = 2) and discarded reads with any ambiguous bases (maxN = 0). We applied a minimum read length of 250 bases after trimming and truncation (minLen = 250). We estimated a parametric error model via a form of unsupervised machine-learning on 100 million sequences for the forward and reverse reads separately. We dereplicated sequencing reads and inferred ASVs for each sample via the DADA2 sample inference algorithm and the estimated error model. We used pseudo-pooling for sample inference to increase sensitivity to ASVs present at low frequencies. We merged denoised sequences using the inferred forward and reverse reads with the length of overlap between forward and reverse reads set to 20 (minOverlap = 20). We allowed for no mismatches in the overlap region (maxMismatch = 0). We identified and removed chimeric sequences via the removeBimeraDenovo function (method = “consensus”). We assigned taxonomy to each of the ASVs using the RDP [9] classifier implementation for DADA2 [10] (taxassignment = ‘rdp’) and SILVA version 138 (reference = SILVA 138; species = SILVA 138) [11]. We removed ASVs classified as Eukaryota and ASVs with unassigned taxonomy at Kingdom-level from the dataset.

**In-silico quality control approach for high quality 16S rRNA gene amplicon sequencing data**

We performed all steps outlined below using R software version 3.6.3 [12] and RStudio version 1.3.1056 [13]. We investigated sequencing reproducibility of bacterial mock community DNA controls by plotting manufacturers’ specified compositions alongside compositions generated in our laboratory. Using within- and between-run repeats we examined sequencing reproducibility of biological specimens in relation to specimen biomass (16S rRNA gene copies/µl), demographic data (participant age at specimen collection) and read counts, as previously described [1]. Next we investigated how profiles from NP specimens compared to profiles from NTCs specimens in relation to specimen biomass, age at specimen collection and read counts, as previously described [1]. We used this information to set cut-offs for excluding NP specimens from downstream analyses based on biomass, age at specimen collection and read counts.

We identified and removed potential “contaminant ASVs” from the dataset by implementing the isContaminant function [14] and a combination of the “frequency- and prevalence-based methods” offered by the *decontam* *package* in R [14]. We removed any remaining “spurious ASV” defined as ASVs with <10 reads across all biological specimens remaining in the dataset [1].

**Statistical analyses**

We investigated associations between covariates and within-specimen (alpha) diversity across three specimen collection intervals [interval A: 1 to 3 months (M01-M03), interval B: 4 to 6 months (M04-M06), and interval C: 7 to 12 months (M07-M12)]. We built Mixed Linear Models (MLMs) to investigate associations between alpha diversity (Shannon diversity index [15] or Chao1 diversity [16]) and covariates via the python package statsmodels version 0.12.2 (https://www.statsmodels.org/stable/index.html). For each target covariate, the fixed effects included the covariate, “timepoint”, and confounders (“age_days_numeric” and “collection_season2”). The random effect (i.e., the “groups” parameter) was the individual participants.

We investigated associations between covariates and between-specimen (beta) diversity at five timepoints (M01, M03, M06, M09 and M12). We used models described by Liu and colleagues [17] to test for differences in beta diversity (Aitchison distance [18, 19] or Bray-Curtis dissimilarity [20]) by covariate. At each timepoint, beta diversity was calculated within each comparison group and between comparison groups for each covariate. All analyses were adjusted for specimen collection season. All p-values were Benjamini-Hochberg (BH) adjusted for false discovery.

We investigated associations between covariates and differential abundance of bacterial genera and amplicon sequence variants (ASVs) across three specimen collection intervals [interval A: 1 to 3 months (M01-M03), interval B: 4 to 6 months (M04-M06), and interval C: 7 to 12 months (M07-M12)]. Only taxa present in more than 90% of the specimens included per specimen collection interval (A, B, or C) were analysed. We performed differential abundance testing using Microbiome Multivariable Associations with Linear Models (MaAsLin2) [21]. We applied a Mixed linear model (mlm) with total sum scaling (TSS) normalization and log transformation, whilst all other parameters set to default. Only taxa with q values <0.10 were deemed differentially abundant. The fixed effects included the covariate, “timepoint”, and confounders (“age_days_numeric” and “collection_season2”). The random effect was the individual participants. By default, ASVs with FDR < 0.25 (BH-corrected P-values) were considered significant by MaAsLin2. In parallel, we also performed differential abundance testing using Analysis of Composition of Microbiomes (ANCOM2) [22] with W-0.6 as the default significance cut-off. We applied a random effects model to each of the three specimen collection intervals. An ANCOM2 detection level ≥0.6 was considered statistically significant and reported if the centered log ratio (clr)-transformed mean difference between groups was >0.03. Both MaAslin2 and ANCOM2 analyses were adjusted for specimen collection age and season.

**Section B: Extended results - sequencing controls and sample selection**

Bacterial profiles from the 24 mock community controls [“BEI-DNA” (n=4) and “Zymobiomics-DNA” (n=20)] included in the five sequencing runs were reproducible and comparable to the theoretical compositions provided by the manufacturer (Figure S2).

**Figure S2 Bacterial profiles sequenced from two sets of bacterial mock community DNA controls**

A) 1-in-10 fold dilutions of HM-783D (BEI Resources, NIAID, NIH as part of the Human Microbiome Project, VA, USA) (“BEI-DNA”), and B) 1-in-10 fold dilutions of ZymoBIOMICS™ Microbial Community DNA Standard (catalogue no. D6305, Zymo Research Corp., Irvine, CA, United States) (“Zymobiomics-DNA”)]. Manufacturers’ specified compositions for each of the bacterial mock community DNA controls are represented by blue circles at the bottom of each of the barplots. Circles in shades of yellow to dark red at the bottom of each of the barplots represent different sequencing runs in which each of the profiles were generated. Taxonomy is assigned at genus-level, with colour-codes representing phylum-level classification (Shades of blue: Proteobacteria, shades of red: Firmicutes). Amplicon sequence variants (ASVs) detected from bacterial mock community DNA profiles which are not assigned to genera as per manufacturers’ specifications are shown in grey.

We observed high sequencing reproducibility from NP specimens randomly selected for repeat processing: “within-run” repeats processed in duplicate (n=20) [median R2 = 0.997 (IQR: 0.988–0.999)] and “between-run repeats” processed in duplicate (n=11), triplicate (n=1) and quadruplicate (n=2) [median R2 = 0.994 (IQR: 0.987–0.998)] across the five runs. NP specimens collected at <10 days of age, NP specimens with <300 16S rRNA gene copies/μl and NP specimens with <1,000 reads were less reproducible compared to NP specimens collected at >10 days of age, NP specimens with >300 16S rRNA gene copies/μl and NP specimens with >1,000 reads, and resultantly flagged for exclusion (Figure S3).

**Figure S3 Associations between reproducibility and A) participant age at specimen collection, B) 16S rRNA gene copy numbers, and C) read counts.**

Reproducibility is measured by coefficient of determination (R^2^) values, calculated by comparing proportions of each amplicon sequence variant (ASV) present between within-run or between-run repeats. Horizontal blue bars denote R^2^ values >0.95. Different shades of vertical blue bars represent A) <10, <50, <100 days of age at the time of specimen collection; B) <1,000, <5,000, <10,000 16S rRNA gene copies/μl; and C) <2,500, <7,500 and <10,000 reads; respectively. For B) and C), each set of “within-run” and “between-run” repeats had two 16S rRNA gene copy number/read count measures shown as two points connected by a horizontal line on the X-axis.

More NP specimens with low biomass (<300 16S rRNA gene copies/μl) were collected at <10 days of age compared to specimens with high biomass (>300 16S rRNA gene copies/μl) (r=0.06) (Figure S4 A). Similarly, more NP specimens with low biomass (<300 16S rRNA gene copies/μl) had lower read counts compared to NP specimens with high biomass (>300 16S rRNA gene copies/μl) (r=0.11) (Figure S4 B). Median read counts from NTCs [7,948 (IQR: 3,372-13,177), n=21] and low biomass NP specimens [7,134 (IQR: 4,828-13,224), n=173] were lower compared to NP specimens with >300 16S rRNA gene copies/μl [21,471 (IQR: 17,315-26,692), n=1,543] (Figure S4 B). More NP specimens with low biomass (<300 16S rRNA gene copies/μl) had higher alpha diversity compared to NP specimens >300 16S rRNA gene copies/μl (r=-0.12) (Figure S4 C). Median alpha diversity indices from NTCs [2.19 (IQR: 1.99-2.56), n=21] and low biomass NP specimens [2.01 (IQR: 1.27-2.79), n=173] were higher compared to median alpha diversity indices from high biomass NP specimens [1.04 (IQR: 0.71-1.40), n=1,543] (Figure S4 C).

**Figure S4** **Participant age at specimen collection, read counts and alpha diversity relative to specimen biomass for no template controls (NTCs) (n = 21) and nasopharyngeal (NP) specimens (n = 1,716)**

A) Scatter plot of participant age at specimen collection, B) read counts following bioinformatic processes, and C) alpha diversity (Shannon diversity) in relation to specimen biomass (16S rRNA gene copies/μl) plotted on log_e_ scale. Vertical orange and blue shaded area highlights <300 and >300 16S rRNA gene copies/μl, respectively.

Lambda scaled [23] logarithm of ratio-transformed data (log-ratio) biplots (incorporating data adjusted in a Bayesian context to remove zeros [18, 19, 24]) showed that NP specimens collected at <10 days of age and NP specimens with <300 16S rRNA gene copies/µl clustered with NTCs (n=21) [1] (Figure S5 A and B). Clustering patterns between NP specimens and NTCs were less evident in relation to read counts (Figure S5 C).

**Figure S5 Two-dimensional dissimilarity plots of beta diversity distance metrices (Aitchison and Bray-Curtis) calculated between specimens in relation to A) participant age at specimen collection, B) 16S rRNA gene copies/μl, and C) read counts following bioinformatic processing**

Logarithm of ratio-transformed (log-ratio) biplots representing dissimilarities between specimens based on Aitchison distance where ASV counts are treated as compositional observations are shown on the left. Principle of coordinate analysis (PCoA) plots representing Bray-Curtis dissimilarities between specimens based on rarefied amplicon sequence variant (ASV) counts are shown on the right. Data points are coloured according to A) participant age at specimen collection (in days), B) 16SrRNA gene copies/μl, and C) read counts available for downstream analyses. Nasopharyngeal (NP) specimens (n = 1,716) are presented by filled circles. No template controls (NTCs) (n = 21) are presented by filled triangles

A total of 1,716 NP specimens (and 3,837 ASVs) from 124 participants were available for downstream analyses. After excluding NP specimens collected at <10 days of age (n=89), remaining NP specimens with <300 16S rRNA gene copies/µl (n=90), and remaining specimens with <1,000 reads (n=1), a total of 1,536 NP specimens were included for downstream analyses. [1] Rarefaction curves following the removal of the 180 NP specimens listed above plateaued at 1,000 reads for most NP specimens (Figure S6).

**Figure S6 Rarefaction curves following the removal of early life and low biomass nasopharyngeal (NP) specimens**

Rarefaction curves represent the number of ASVs detected in relation to the read counts obtained from each specimen. The dotted vertical line denotes 1,000 reads. Rarefaction curves are coloured according to A) age at specimen collection, B) specimen biomass (16S rRNA gene copies/μl) and C) read counts.

We further removed 147 (5%) of the remaining 2,851 ASVs, identified as potential contaminants [1, 14]. Potential contaminant ASVs were detected at low prevalence in NP specimens [median: 7 (IQR: 3-19)]. We then removed 1,505 (58%) of the remaining 2,704 ASVs identified as “spurious ASVs” (ASVs with <10 reads across the dataset). “Spurious ASVs” represented <0.002% of the sequencing profile in any given NP specimen. We excluded an additional 21 participants (178 NP specimens and 48 ASVs) for reasons outlined in Figure S7.

**Figure S7** **Nasopharyngeal (NP) specimen and participant selection for downstream analyses**

A) 1,716 NP specimens (and 3,837 amplicon sequence variants (ASVs)) from 124 participants were available following bioinformatic processing; B) 1,536 NP specimens (and 1,079 ASVs) were available following the exclusion of NP specimens and ASVs by applying a step-wise in-silico quality control approach [1] to ensure the inclusion of high quality gene amplicon data; C) 1,358 NP specimens (and 1,031 ASVs) from 103 participants were included to study NP bacterial profiles over time; D) 927 NP specimens (and 682 ASVs) from 99 participants were included to investigate associations between NP bacterial profiles and exposures in the first year of life.

**Section C: Extended results – Figures S8 – S19**

**Figure S8** Compositional biplot of the subject loadings from Compositional Tensor Factorization (CTF) analysis, showing the top two ordination axes. Each point represents the time series of a participant, and arrows represent the amplicon sequence variants (ASVs) differentiating between participants. Numbers refer to ASVs, e.g., 1 refers to ASV_1.

**
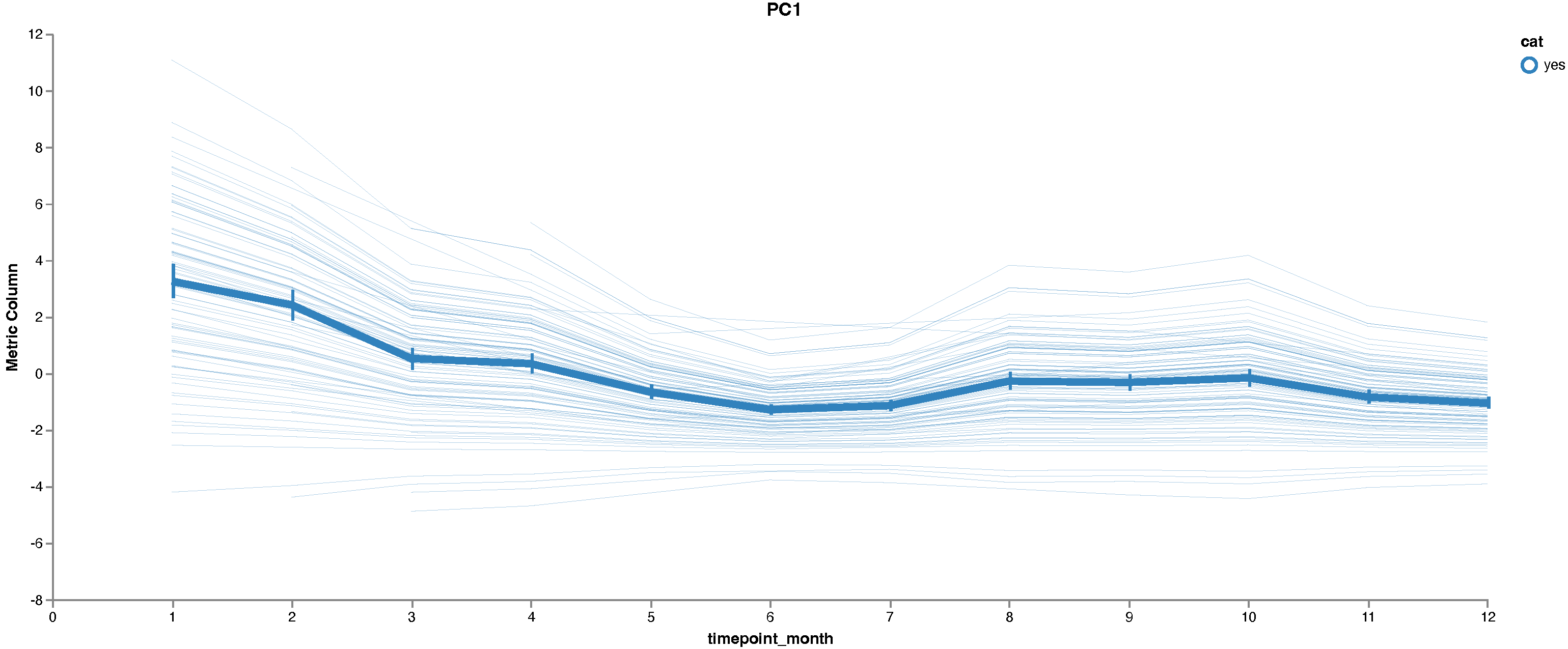
**

**
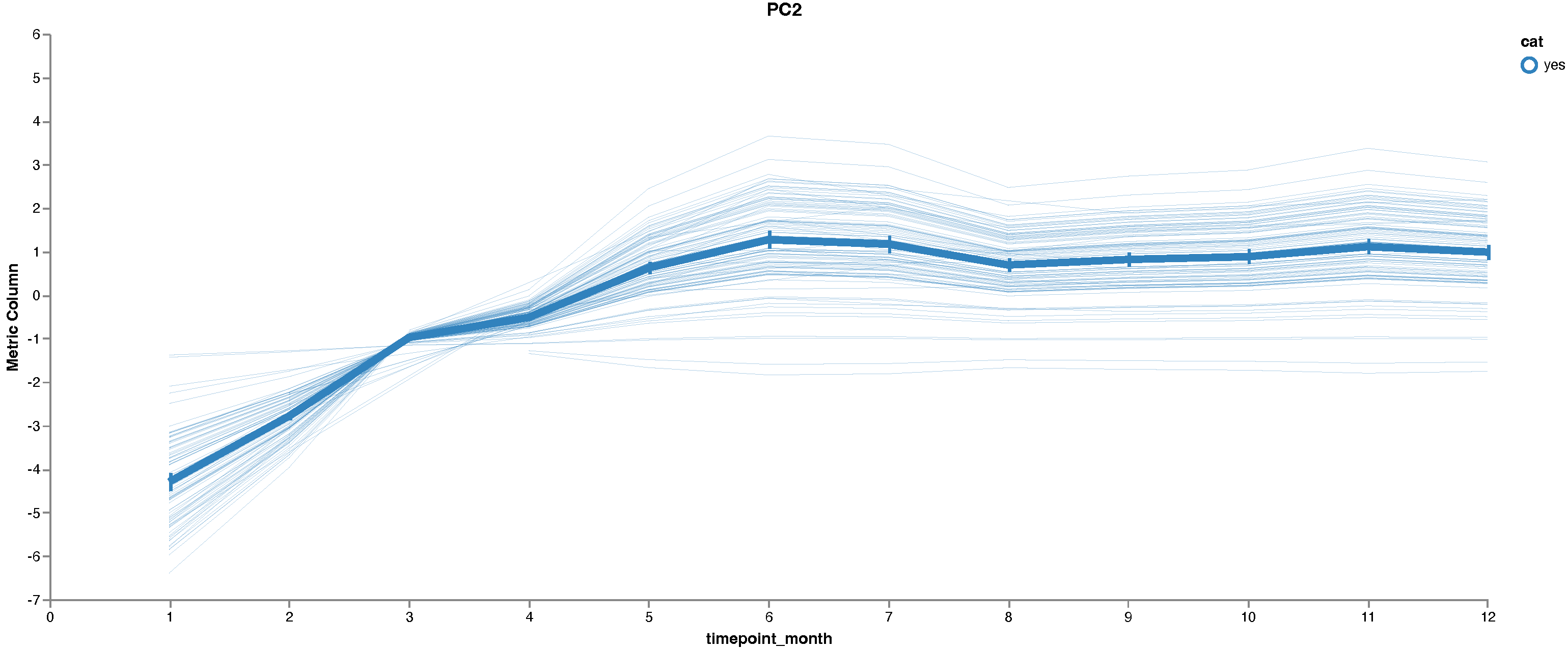
**

**Figure S9** Trajectory analysis of the two top ordination axes (PC1 and PC2) derived from Compositional Tensor Factorization (CTF analysis). Each thin line represents an individual participant, while the bold line represents the mean trajectory for all participants.

**Figure S10** **Nasopharyngeal (NP) bacterial composition of most abundant genera detected at each timepoint from each of the 103 participants during the first 30 months of life.**

Each row represents a participant, sampled over time. Participants are grouped on the Y-axis according to the bacterial genus with the highest relative abundance detected at one month of life (M01): A) *Corynebacterium*; B) *Staphylococcus*; C) *Moraxella*; D) *Haemophilus*; E) *Streptobacillus*; F) *Klebsiella*; G) *Neisseria*; H) *Streptococcus;* I) *Dolosigranulum*. Participants with missing specimens at M01 are grouped at the bottom of the plot (J). Specimen collection age (in months) and the number of specimens analyzed at each timepoint are shown on the X-axis. Relative abundances of the most abundant bacterial genera that make up 90% of each specimen profile (maximum of 10 bacterial genera per specimen profile plot) are represented using colours corresponding to the phyla to which they belong [shades of blue: Proteobacteria (and Campilobacterota: *Campylobacter* and *ASV_136*), shades of yellow: Actinobacteria, shades of red: Firmicutes, shades of pink: Fusobacteriota, shades of green: Bacteroidetes, shades of purple: Verrucomicrobiota]. Grey bars represent all other bacterial genera detected from each of the specimens. Blank/white spaces represent missing specimens at each timepoint.

**Figure S11** **Within-specimen (Shannon) diversity measured from comparison groups during the first year of life.**

Boxplots represent Shannon diversity indices measured from specimens at monthly intervals during the first year of life. Specimen collection timepoints are grouped into three intervals (interval A: M01-M03, interval B: M04-M06, and interval C: M07-M12). Mixed Linear Models (MLMs) were built for each of the three specimen collection intervals to investigate associations between Shannon diversity and covariates. Median values are presented by horizontal lines within each of the boxplots while upper and lower ranges of the boxplots represent the 75% and 25% quartiles, respectively. Maximum and minimum values, excluding outliers, are presented by whiskers. Dots at each of the timepoints represent mean values. Each connecting line between boxplots within a specimen collection interval represents changes in Shannon diversity indices for each of the participants. All p-values were adjusted for confounders. Statistically significant p-values are shown presented in red font.

**Figure S12 Between-specimen (Aitchison distance) diversity measured within and between covariate groups at five timepoints during the first year of life.**

Boxplots represent beta diversity (Aitchison distance) measured between specimens at five timepoints (M01, M03, M06, M09 and M12). Boxplots colors correspond to covariate groups. Each color-filled boxplot represents pairwise specimen beta diversity within the covariate level (within-level beta diversity). Each grey filled boxplot represents pairwise specimen beta diversity between different groups of the covariate tested (between-level beta diversity). Each grey boxplot has two dots above and below the median which correspond to the groups for which beta diversity is being compared. Median values are presented by horizontal lines within each of the boxplots while upper and lower ranges of the boxplots represent the 75% and 25% quartiles, respectively. Maximum and minimum values, excluding outliers, are presented by whiskers. All p-values were adjusted for confounders. P-values in brackets were corrected using the Benjamini-Hochberg (BH) adjustment for false discovery rate. Statistically significant p-values are presented in red font.

**Figure S13 Between-specimen (Bray-Curtis dissimilarity) diversity measured within and between covariate groups at five timepoints during the first year of life.**

Boxplots represent beta diversity (Bray-Curtis dissimilarity) measured between specimens at five timepoints (M01, M03, M06, M09 and M12). Boxplots colors correspond to covariate groups. Each color-filled boxplot represents pairwise specimen beta diversity within the covariate level (within-level beta diversity). Each grey filled boxplot represents pairwise specimen beta diversity between different groups of the covariate tested (between-level beta diversity). Each grey boxplot has two dots above and below the median which correspond to the groups for which beta diversity is being compared. Median values are presented by horizontal lines within each of the boxplots while upper and lower ranges of the boxplots represent the 75% and 25% quartiles, respectively. Maximum and minimum values, excluding outliers, are presented by whiskers. All p-values were adjusted for confounders. P-values in brackets were corrected using the Benjamini-Hochberg (BH) adjustment for false discovery rate. Statistically significant p-values are presented in red font.

**Figure S14** **Within-specimen (Shannon) diversity measured from comparison groups during the first year of life.**

Boxplots represent Shannon diversity indices measured from specimens at monthly intervals during the first year of life. Specimen collection timepoints are grouped into three intervals (interval A: M01-M03, interval B: M04-M06, and interval C: M07-M12). Mixed Linear Models (MLMs) were built for each of the three specimen collection intervals to investigate associations between Shannon diversity and covariates. Median values are presented by horizontal lines within each of the boxplots while upper and lower ranges of the boxplots represent the 75% and 25% quartiles, respectively. Maximum and minimum values, excluding outliers, are presented by whiskers. Dots at each of the timepoints represent mean values. Each connecting line between boxplots within a specimen collection interval represents changes in Shannon diversity indices for each of the participants. All p-values were adjusted for confounders. Statistically significant p-values are shown presented in red font.

**Figure S15 Between-specimen (Aitchison distance) diversity measured within and between covariate groups at five timepoints during the first year of life.**

Boxplots represent beta diversity (Aitchison distance) measured between specimens at five timepoints (M01, M03, M06, M09 and M12). Boxplots colors correspond to covariate groups. Each color-filled boxplot represents pairwise specimen beta diversity within the covariate level (within-level beta diversity). Each grey filled boxplot represents pairwise specimen beta diversity between different groups of the covariate tested (between-level beta diversity). Each grey boxplot has two dots above and below the median which correspond to the groups for which beta diversity is being compared. Median values are presented by horizontal lines within each of the boxplots while upper and lower ranges of the boxplots represent the 75% and 25% quartiles, respectively. Maximum and minimum values, excluding outliers, are presented by whiskers. All p-values were adjusted for confounders. P-values in brackets were corrected using the Benjamini-Hochberg (BH) adjustment for false discovery rate. Statistically significant p-values are presented in red font.

**Figure S16 Between-specimen (Bray-Curtis dissimilarity) diversity measured within and between covariate groups at five timepoints during the first year of life.**

Boxplots represent beta diversity (Bray-Curtis dissimilarity) measured between specimens at five timepoints (M01, M03, M06, M09 and M12). Boxplots colors correspond to covariate groups. Each color-filled boxplot represents pairwise specimen beta diversity within the covariate level (within-level beta diversity). Each grey filled boxplot represents pairwise specimen beta diversity between different groups of the covariate tested (between-level beta diversity). Each grey boxplot has two dots above and below the median which correspond to the groups for which beta diversity is being compared. Median values are presented by horizontal lines within each of the boxplots while upper and lower ranges of the boxplots represent the 75% and 25% quartiles, respectively. Maximum and minimum values, excluding outliers, are presented by whiskers. All p-values were adjusted for confounders. P-values in brackets were corrected using the Benjamini-Hochberg (BH) adjustment for false discovery rate. Statistically significant p-values are presented in red font.

**Figure S17** **Within-specimen (Shannon) diversity measured from comparison groups during the first year of life.**

Boxplots represent Shannon diversity indices measured from specimens at monthly intervals during the first year of life. Specimen collection timepoints are grouped into three intervals (interval A: M01-M03, interval B: M04-M06, and interval C: M07-M12). Mixed Linear Models (MLMs) were built for each of the three specimen collection intervals to investigate associations between Shannon diversity and covariates. Median values are presented by horizontal lines within each of the boxplots while upper and lower ranges of the boxplots represent the 75% and 25% quartiles, respectively. Maximum and minimum values, excluding outliers, are presented by whiskers. Dots at each of the timepoints represent mean values. Each connecting line between boxplots within a specimen collection interval represents changes in Shannon diversity indices for each of the participants. All p-values were adjusted for confounders. Statistically significant p-values are shown presented in red font.

**Figure S18 Between-specimen (Aitchison distance) diversity measured within and between covariate groups at five timepoints during the first year of life.**

Boxplots represent beta diversity (Aitchison distance) measured between specimens at five timepoints (M01, M03, M06, M09 and M12). Boxplots colors correspond to covariate groups. Each color-filled boxplot represents pairwise specimen beta diversity within the covariate level (within-level beta diversity). Each grey filled boxplot represents pairwise specimen beta diversity between different groups of the covariate tested (between-level beta diversity). Each grey boxplot has two dots above and below the median which correspond to the groups for which beta diversity is being compared. Median values are presented by horizontal lines within each of the boxplots while upper and lower ranges of the boxplots represent the 75% and 25% quartiles, respectively. Maximum and minimum values, excluding outliers, are presented by whiskers. All p-values were adjusted for confounders. P-values in brackets were corrected using the Benjamini-Hochberg (BH) adjustment for false discovery rate. Statistically significant p-values are presented in red font.

**Figure S19 Between-specimen (Bray-Curtis dissimilarity) diversity measured within and between covariate groups at five timepoints during the first year of life.**

Boxplots represent beta diversity (Bray-Curtis dissimilarity) measured between specimens at five timepoints (M01, M03, M06, M09 and M12). Boxplots colors correspond to covariate groups. Each color-filled boxplot represents pairwise specimen beta diversity within the covariate level (within-level beta diversity). Each grey filled boxplot represents pairwise specimen beta diversity between different groups of the covariate tested (between-level beta diversity). Each grey boxplot has two dots above and below the median which correspond to the groups for which beta diversity is being compared. Median values are presented by horizontal lines within each of the boxplots while upper and lower ranges of the boxplots represent the 75% and 25% quartiles, respectively. Maximum and minimum values, excluding outliers, are presented by whiskers. All p-values were adjusted for confounders. P-values in brackets were corrected using the Benjamini-Hochberg (BH) adjustment for false discovery rate. Statistically significant p-values are presented in red font.

**Table S5 See separate spreadsheet.**

**Section D: Extended references**

1. Claassen-Weitz S, Gardner-Lubbe S, Mwaikono KS, du Toit E, Zar HJ, Nicol MP. Optimizing 16S rRNA gene profile analysis from low biomass nasopharyngeal and induced sputum specimens. BMC Microbiol. 2020;20:113.

2. Claassen-Weitz S, Gardner-Lubbe S, Nicol P, Botha G, Mounaud S, Shankar J, et al. HIV-exposure, early life feeding practices and delivery mode impacts on faecal bacterial profiles in a South African birth cohort. Sci Rep. 2018;8:1–15. doi:10.1038/s41598-018-22244-6.

3. Illumina Proprietary. MiSeq ® Reagent Kit v3 Reagent Preparation Guide. 2013; October 2013:1–14. http://supportres.illumina.com/documents/documentation/system_documentation/miseq/miseq-reagent-kit-v3-reagent-prep-guide-15044983-b.pdf.

4. Illumina Proprietary. MiSeq ® System User Guide. 2014; January:1–94. http://supportres.illumina.com/documents/documentation/system_documentation/miseq/miseq-system-user-guide-15027617-n.pdf.

5. Andrews S. FastQC: a quality control tool for high throughput sequence data. 2010. http://www.bioinformatics.babraham.ac.uk/projects/fastqc.

6. Ewels P, Magnusson M, Lundin S, Käller M. MultiQC: Summarize analysis results for multiple tools and samples in a single report. Bioinformatics. 2016;32:3047–8.

7. Callahan BJ, McMurdie PJ, Rosen MJ, Han AW, Johnson, Amy Jo A Holmes SP. DADA2: High resolution sample inference from Illumina amplicon data. Nat Methods. 2016;13:581–3.

8. Tommaso P Di, Chatzou M, Floden EW, Barja PP, Palumbo E, Notredame C. Nextflow enables reproducible computational workflows. Nat Biotechnol. 2017;35:316–9.

9. Cole JR, Wang Q, Fish JA, Chai B, McGarrell DM, Sun Y, et al. Ribosomal Database Project: Data and tools for high throughput rRNA analysis. Nucleic Acids Res. 2014;42:633–42.

10. Callahan BJ. RDP taxonomic training data formatted for DADA2 (RDP trainset 16/release 11.5). Zenodo. 2017. https://zenodo.org/record/801828#.X7VWKs7itdg.

11. Quast C, Pruesse E, Yilmaz P, Gerken J, Schweer T, Yarza P, et al. The SILVA ribosomal RNA gene database project: improved data processing and web-based tools. Nucleic Acids Res. 2013;41 Database issue:D590-6. doi:10.1093/nar/gks1219.

12. R Core Team. R Foundation for Statistical Computing. R: A language and environment for statistical computing. 2021. https://www.r-project.org/.

13. RStudio Team. RStudio: Integrated development environment for R. 2021. http://www.rstudio.org/.

14. Davis NM, Proctor D, Holmes SP, Relman DA, Callahan BJ. Simple statistical identification and removal of contaminant sequences in marker-gene and metagenomics data. bioRxiv. 2017;:221499. doi:10.1101/221499.

15. Shannon CE. A Mathematical Theory of Communication. Bell Syst Tech J. 1948;27:379–423.

16. Chao A. Estimating the Population Size for Capture-Recapture Data with Unequal Catchability. Biometrics. 1987;43:783–91.

17. Liu J, Zhang X, Chen T, Wu T, Lin T, Jiang L, et al. A semiparametric model for between-subject attributes: Applications to beta-diversity of microbiome data. Biometrics. 2021; May:1–13.

18. Aitchison J. The Statistical Analysis of Compositional Data. J R Stat Soc. 1982;44:139–60.

19. Aitchison J, Greenacre M. Biplots of compositional data. J R Stat Soc Ser C (Applied Stat. 2002;51:375–92.

20. Bray JR, Curtis JT. An Ordination of the Upland Forest Communities of Southern Wisconsin. Ecol Monogr. 1957;27:325–49.

21. Mallick H, Rahnavard A, McIver LJ, Ma S, Zhang Y, Nguyen LH, et al. Multivariable association discovery in population-scale meta-omics studies. PLoS Comput Biol. 2021;17:1–27. doi:10.1371/journal.pcbi.1009442.

22. Mandal S, Van Treuren W, White RA, Eggesbø M, Knight R, Peddada SD. Analysis of composition of microbiomes: a novel method for studying microbial composition. Microb Ecol Heal Dis. 2015;26:27663.

23. Gower J, Lubbe S, Le Roux N. Understanding Biplots. Chichester, United Kingdom: John Wiley & Sons Ltd.; 2011.

24. Martin-Fernandez J., Palarea-Albaladejo J, Olea R. Dealing with Zeros. In: Pawlowsky-Glahn V, Buccianti A, editors. Compositional Data Analysis: Theory and Applications. Chichester, United Kingdom: John Wiley & Sons Ltd.; 2011. p. 43–58.
